# Pitaya‑inspired compartmentalized microspheres with natural tannic acid-copper coating orchestrate smart release of ions and multi-drugs for synergistic treatment of infected bone defects

**DOI:** 10.1093/rb/rbag101

**Published:** 2026-05-27

**Authors:** Dongbiao Chang, Zhenfan Bai, Linke Li, Jun Sheng, Hongzhi Fang, Zian Wang, Zili Guo, Hui Zhang, Xinxi Yang, Rui Chen, Huan Tan, Mengyuan Wang, Jie Weng

**Affiliations:** Institute of Biomedical Engineering, College of Medicine, Southwest Jiaotong University, Chengdu 610031, China; Key Laboratory of Advanced Technologies of Materials Ministry of Education, School of Materials Science and Engineering, Southwest Jiaotong University, Chengdu 610031, China; State Key Laboratory of Oral Diseases and National Center for Stomatology, National Clinical Research Center for Oral Diseases, West China Hospital of Stomatology, Sichuan University, Chengdu 610041, China; The Center of Obesity and Metabolic Diseases, Department of General Surgery, The Third People’s Hospital of Chengdu & The Affiliated Hospital of Southwest Jiaotong University, Chengdu 610014, China; Institute of Biomedical Engineering, College of Medicine, Southwest Jiaotong University, Chengdu 610031, China; Key Laboratory of Advanced Technologies of Materials Ministry of Education, School of Materials Science and Engineering, Southwest Jiaotong University, Chengdu 610031, China; Institute of Biomedical Engineering, College of Medicine, Southwest Jiaotong University, Chengdu 610031, China; The Center of Obesity and Metabolic Diseases, Department of General Surgery, The Third People’s Hospital of Chengdu & The Affiliated Hospital of Southwest Jiaotong University, Chengdu 610014, China; Department of Orthopedic, The General Hospital of Western Theater Command of PLA, Chengdu 610083, China; The Center of Obesity and Metabolic Diseases, Department of General Surgery, The Third People’s Hospital of Chengdu & The Affiliated Hospital of Southwest Jiaotong University, Chengdu 610014, China; Institute of Biomedical Engineering, College of Medicine, Southwest Jiaotong University, Chengdu 610031, China; Key Laboratory of Advanced Technologies of Materials Ministry of Education, School of Materials Science and Engineering, Southwest Jiaotong University, Chengdu 610031, China; Institute of Biomedical Engineering, College of Medicine, Southwest Jiaotong University, Chengdu 610031, China; Key Laboratory of Advanced Technologies of Materials Ministry of Education, School of Materials Science and Engineering, Southwest Jiaotong University, Chengdu 610031, China; Institute of Biomedical Engineering, College of Medicine, Southwest Jiaotong University, Chengdu 610031, China; Key Laboratory of Advanced Technologies of Materials Ministry of Education, School of Materials Science and Engineering, Southwest Jiaotong University, Chengdu 610031, China; The Center of Obesity and Metabolic Diseases, Department of General Surgery, The Third People’s Hospital of Chengdu & The Affiliated Hospital of Southwest Jiaotong University, Chengdu 610014, China; Institute of Biomedical Engineering, College of Medicine, Southwest Jiaotong University, Chengdu 610031, China; The Center of Obesity and Metabolic Diseases, Department of General Surgery, The Third People’s Hospital of Chengdu & The Affiliated Hospital of Southwest Jiaotong University, Chengdu 610014, China; Institute of Biomedical Engineering, College of Medicine, Southwest Jiaotong University, Chengdu 610031, China; The Center of Obesity and Metabolic Diseases, Department of General Surgery, The Third People’s Hospital of Chengdu & The Affiliated Hospital of Southwest Jiaotong University, Chengdu 610014, China; Institute of Biomedical Engineering, College of Medicine, Southwest Jiaotong University, Chengdu 610031, China; State Key Laboratory of Oral Diseases and National Center for Stomatology, National Clinical Research Center for Oral Diseases, West China Hospital of Stomatology, Sichuan University, Chengdu 610041, China; Institute of Biomedical Engineering, College of Medicine, Southwest Jiaotong University, Chengdu 610031, China; Key Laboratory of Advanced Technologies of Materials Ministry of Education, School of Materials Science and Engineering, Southwest Jiaotong University, Chengdu 610031, China

**Keywords:** compartmentalized microspheres, spatiotemporally pH-responsive release, antibacterial activity, reprogrammed macrophage, infected bone defect repair

## Abstract

Infected bone defects present a significant clinical challenge, as persistent bacterial burden and exacerbated inflammatory response induce microenvironmental acidification, which severely compromises tissue regeneration. Therefore, this study aims to construct a smart platform to regulate the local microenvironment and achieve the therapeutic effect of bone regeneration. Here, pitaya‑inspired compartmentalized microspheres (IBPR‑CuTA) were developed to provide spatiotemporally controlled pH‑responsive release of Cu^2+^, antibiotic rifampicin (RF) and natural extract icariin (ICA), coupling infection control with tissue regeneration. Specifically, under infected inflammation, the microspheres rapidly release Cu^2+^ and RF, which facilitates early anti-bacterial and anti-inflammatory effects and promotes pH normalization. This is succeeded by a delayed release of remaining Cu^2+^ to foster angiogenesis, and a subsequent sustained release of encapsulated RF and ICA, collectively providing long-term inhibition of infection recurrence and promotion of bone regeneration. Mechanistically, IBPR‑CuTA was associated with suppressed glucose metabolism-related signaling and TNF/NF‑κB activation, and induced a shift from pro‑inflammatory M1 to reparative M2 polarization. *In vivo*, IBPR‑CuTA concurrently eradicated infection and accelerated vascularized bone regeneration in an infected defect model. Collectively, these results demonstrate an immunometabolically guided, spatiotemporally programmed microsphere platform that integrates early bactericidal/antioxidant activity with sustained pro‑regenerative cues, highlighting translational potential for managing complex infected bone defects.

## Graphical abstract

**Graphical Abstract:**
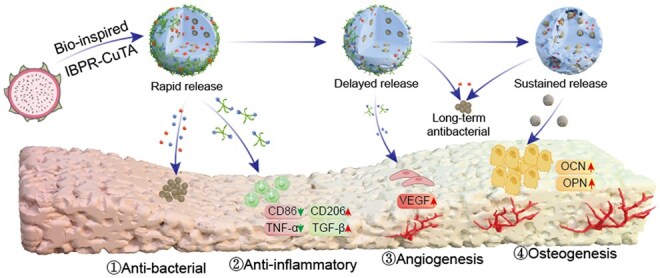

## Introduction

With the acceleration of global population aging and the rising burden of orthopedic diseases, infected bone defects have emerged as a formidable clinical challenge. Epidemiological data indicate that the incidence of bone infection in patients with open fractures can reach up to 33%, with treatment failure rates between 10% and 40% [1–3]. Unlike ordinary bone defects, infected bone defects are often characterized by a dysregulated immune microenvironment and disordered bone metabolism, and frequently involve necrotic bone tissue [4]. The condition can further progress to chronic osteomyelitis with a high risk of relapse, significantly prolonging the treatment course and increasing the financial burden of patients [5, 6]. The combination of persistent infection and impaired healing makes such defects particularly difficult to manage in orthopedic practice.

Current management of infected bone defects centers on meticulous debridement with local or systemic antibiotic coverage to suppress infection, followed by staged repair and reconstruction [7]. Nevertheless, conventional strategies often fail to satisfy the three imperatives of infection control, immunomodulation and bone regeneration simultaneously. Effective therapy requires complete removal of necrotic tissue at the lesion, eradication of bacteria and suppression of subsequent regrowth [8, 9]. It also requires remodeling of the post-infectious immune response, particularly directing macrophage polarization away from the pro-inflammatory M1 state toward a reparative M2 phenotype to establish an immune milieu conducive to tissue renewal [2, 10]. In parallel, angiogenesis must be promoted within the defect to secure oxygen and nutrient delivery and to re-establish a regenerative niche for bone healing. The intricate nature of these simultaneous demands presents a formidable challenge that current treatments are ill-equipped to address, resulting in a substantial unmet clinical deficiency.

To address this challenge, multifunctional biomaterials have offered new avenues for comprehensive infection control-immunomodulation-regeneration therapy [11, 12]. For example, the antibiotic rifampicin (RF) exhibits potent bactericidal activity against *Staphylococcus aureus*, and combining it with other antimicrobial agents can reduce the risk of resistance [13, 14]. Meanwhile, copper ions (Cu^2+^) can directly kill bacteria by disrupting their cell membranes, which not only enhances antibiotic efficacy but also helps suppress the emergence of resistant strains [15, 16]. Beyond direct antibacterial effects, incorporating immunomodulatory and pro-regenerative elements is equally critical. The copper-tannic acid (CuTA) coordination network has attracted attention for its facile synthesis and good biocompatibility [17, 18]. Such materials not only provide broad-spectrum antibacterial activity but also display significant reactive oxygen species (ROS) scavenging ability, suppressing the production of pro-inflammatory factors and inducing macrophage polarization from the pro-inflammatory M1 state toward the reparative M2 phenotype [19, 20]. In parallel, natural bioactive molecules have shown potential in stimulating tissue regeneration. For instance, icariin (ICA) possesses antioxidant, anti-inflammatory and osteogenic activities [21–23]. Furthermore, its combination with biphasic calcium phosphate (BCP) has been shown to significantly promote new bone formation [24, 25]. Therefore, rationally integrating these antibiotics, immunomodulatory factors, and osteogenic components into a single platform offers a promising strategy to address the complex pathology of infected bone defects.

Beyond the composition of therapeutic agents, another key determinant of efficacy is the mode of drug delivery. Local delivery (either *in situ* release or via a local carrier) can achieve high drug concentrations at the lesion while reducing systemic side effects. While pH-responsive drug delivery systems are a recognized and maturing field, achieving truly precise spatiotemporal control over multi-drug release for complex pathologies like infected bone defects remains a significant challenge. For example, in clinical practice, antibiotic-loaded polymethyl methacrylate (PMMA) bone cement can temporarily suppress infection, but 80–90% of its drug payload is released in a burst within minutes, meaning the duration of effective drug levels is far shorter than intended [26, 27]. Biodegradable polymer carriers such as poly (lactic-co-glycolic acid) (PLGA) microspheres have good injectability and have been widely used in tissue fillers and controlled-release systems [28–30]. However, existing microspheres or scaffolds mostly focus on one or a few antibiotics, often neglecting other bioactive cues that are equally crucial for tissue repair [31, 32]. Moreover, the highly hydrophobic surface of PLGA hinders cell adhesion and proliferation, weakening cell-material interactions and potentially affecting cell survival and function [33].

Recent advancements have explored biomaterials that integrate some functionalities, including multi-shell or core-shell structured materials, which offer distinct advantages for encapsulating diverse therapeutic agents and enabling programmed, multi-stage release kinetics [34, 35]. However, many of these existing systems still grapple with optimizing the delicate balance between rapid infection control, fine-tuned immune modulation and robust tissue regeneration within a single, coherent design. They often lack the sophisticated hierarchical architecture required to orchestrate multiple therapeutic actions synergistically and sequentially. These limitations underscore the urgent need for a delivery platform that enables multi-drug co-loading, is disease-responsive, provides sequential release and actively modulates cell fate, ultimately bridging the gap between theoretical potential and practical clinical efficacy.

**Figure 1:**
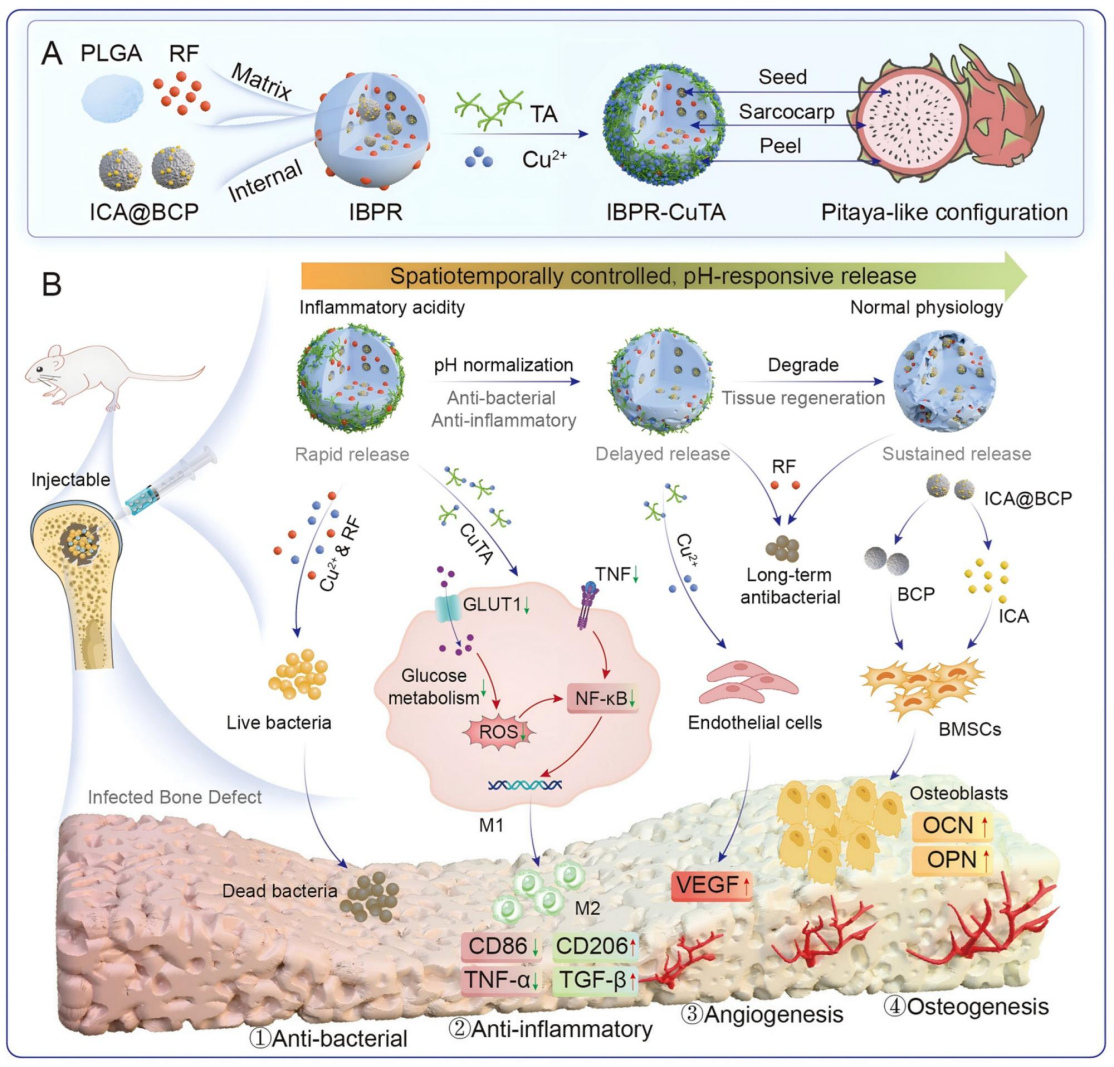
Illustration of constructing the IBPR-CuTA microspheres with the pitaya-like configuration and their application for the repair of infected bone defects. (**A**) Compartmentalized microspheres were constructed with ICA@BCP particles distributed within internal compartments and an RF‑loaded PLGA matrix. Subsequently, a CuTA coating was formed on the microsphere surface. This composite architecture resembled a pitaya‑like configuration, in which ICA@BCP particles as seeds were embedded in the sarcocarp, which was composed of RF-encapsulated PLGA and enclosed with CuTA as the peel. (**B**) Upon injection into the infected defect, IBPR‑CuTA microspheres provide spatiotemporally controlled pH‑responsive release of Cu^2+^, RF and ICA. An initial rapid, pH-sensitive release of Cu^2+^ and RF enables potent antibacterial activity and macrophage metabolic reprogramming-driven anti-inflammatory effects, leading to pH normalization. This is followed by a delayed release of remaining Cu^2+^ to foster angiogenesis, and subsequently, a sustained release of encapsulated RF and ICA, collectively providing long-term inhibition of infection recurrence and promoting osteogenic functions, thus facilitating the effective repair of infected bone defects.

Based on these considerations, we developed a multifunctional microsphere strategy that combined deliberate materials design with immunomodulatory and osteogenic synergy. Building upon the principles of advanced multi-shell material design and addressing the aforementioned critical limitations, we constructed a compartmentalized microsphere designed for spatiotemporally controlled pH‑responsive release, termed IBPR‑CuTA, with a pitaya-inspired configuration in which seed-like compartments are embedded in a sarcocarp-like matrix and enclosed by a peel-like outer layer. This hierarchical and multi-compartmental architecture provides a strategy for spatially organizing different therapeutic components and supporting their temporally regulated release under infection-related acidic conditions. Through this refined structure, IBPR-CuTA achieved pH-responsive, multi-stage sequential release under acidic inflammatory conditions: it orchestrated an initial release of Cu^2+^ and RF for early infection control, followed by sustained release of RF and ICA over an extended period to support the re-establishment of immune homeostasis and bone regeneration, in a manner responsive to the local inflammatory pH. In this platform, the internally compartmentalized ICA@BCP particles provided osteogenic cues, the sarcocarp-like PLGA encapsulated RF for localized antibacterial action, and the outermost CuTA coordination network coating conferred immunomodulatory functionality along with additional antibacterial activity. *In vitro* studies confirmed that IBPR‑CuTA exhibited highly efficient and long-lasting antibacterial activity. In macrophage co-culture, the microspheres effectively scavenged intracellular ROS and reprogrammed macrophages from the M1 toward M2 by suppressing glucose metabolism and the TNF/NF‑κB signaling pathway, thereby exhibiting pronounced immunomodulatory activity. Building on the reshaped immune microenvironment, IBPR‑CuTA further enhanced angiogenesis and osteogenic differentiation. In an infected bone defect model, IBPR‑CuTA effectively eradicated *S.aureus* infection and significantly accelerated bone tissue regeneration (Figure 1). Taken together, this study provided a comprehensive solution for treating infected bone defects and illustrated how a strategically structured multifunctional microsphere achieved robust infection control, precise immune modulation and enhanced bone regeneration within a single platform. The key advantages of our IBPR-CuTA system, stemming directly from its innovative multi-compartmental architecture, include: (i) a novel and sophisticated design that enables precise spatiotemporal control over drug release kinetics, (ii) the synergistic integration of broad-spectrum antibacterial agents (Cu^2+^ and RF) with immunomodulatory (CuTA) and pro-osteogenic/angiogenic cues (ICA and BCP) within distinct, functionally optimized compartments; and (iii) its inherent pH-responsive behavior, allowing for targeted activation and an orchestrated sequence of therapeutic actions at infection sites. These combined features may offer a coordinated multifunctional strategy for infected bone defect repair and provide a useful design framework for future local delivery systems.

## Materials and methods

### Materials

Calcium nitrate, disodium hydrogen phosphate, nitric acid, urea, anhydrous ethanol, dichloromethane, polyvinyl alcohol (PVA), copper chloride and tannic acid (TA) were provided by Cologne Chemicals (Chengdu, China). 1,2,3,4,5,6-Cyclohexanehexacarboxylic acid monohydrate (H6L), ICA, RF and glutaraldehyde were provided by Aladdin (Shanghai, China). PLGA (LA: GA = 50:50, MW = 30 kDa) was supplied by Daigang Biomaterial Co., Ltd. (Jinan, China).

### Preparation of compartmentalized IBPR-CuTA microspheres

#### Preparation of ICA@BCP

First, a calcium phosphate (CaP) solution was prepared with a Ca^2+^ concentration of 0.06 M and a Ca:P molar proportion set at 1.67. Nitric acid was used to clarify the solution, followed by the addition of H6L and urea under continuous stirring. After autoclave treatment at 95°C for 5 h, the resulting precipitate was collected and calcined at 800°C for 2 h to yield BCP microspheres. The above experimental conditions and parameters were obtained through optimization of the multi-channel parallel hydrothermal synthesis process [36, 37]. Subsequently, 50 mg of the BCP microspheres were mixed with an ICA solution (2 mg/mL) for 2 h. Unloaded ICA was removed, and after freeze-drying, ICA@BCP was obtained.

#### Preparation of IBPR microspheres

An amount of 100 mg of PLGA was dissolved in 3 mL dichloromethane (oil phase, O), followed by 1.5 mg RF and 50 mg ICA@BCP (solid phase, S). The mixture was thoroughly blended to obtain a solid-in-oil (S/O) suspension. The S/O mixture was then added dropwise into 50 mL of PVA solution (water phase, W) and stirred at 400 rpm to form a solid-in-oil-in-water (S/O/W) emulsion. After complete evaporation of dichloromethane, PLGA microspheres co-encapsulating RF and ICA@BCP (IBPR) were collected. Similarly, PLGA microspheres loaded with ICA@BCP (IBP) were fabricated using the identical method, except without the addition of RF.

#### Preparation of CuTA coating on compartmentalized microspheres

The IBPR microspheres were immersed in a 2-mg/mL TA solution (pH 8.0) and stirred overnight. After washing, the microspheres were transferred to a 5-mmol/L copper chloride solution for 2 h. Finally, IBPR-CuTA was collected after freeze-drying.

### Characterizations

The injectability of the microspheres was evaluated using a 23-G needle. The microspheres were mixed with an appropriate amount of saline and loaded into a syringe, and their extrusion performance was observed. To assess the degradation and release profiles of the microspheres, IBPR-CuTA was immersed separately in PBS. On Days 1, 3, 7, 14, 21 and 28, the microspheres were collected, freeze-dried and weighed to evaluate their remaining mass, which reflected the degradation rate. The release curves of ICA and RF were determined by monitoring their absorbance using a Ultraviolet (UV)-visible spectrophotometer (UH4150, Hitachi, Japan).

1,1-Diphenyl-2-picrylhydrazyl (DPPH) and 2,2'-azino-bis (3-ethylbenzothiazoline-6-sulfonic acid) (ABTS) assays were employed to evaluate the antioxidant activity of IBPR-CuTA. According to previous report [38], IBPR-CuTA was incubated separately with DPPH and ABTS. Absorbance and radical scavenging activities were subsequently assessed.

### *In vitro* biocompatibility assessment

The hemolysis rate was determined to evaluate the hemocompatibility of the materials. For controls, red blood cells without material addition constituted the negative group, and those resuspended in deionized water comprised the positive group. Following a 1-h incubation of the samples with red blood cells at 37°C, the extent of hemolysis was evaluated. Subsequently, the supernatant was separated and used to calculate the hemolysis rate.

MC3T3-E1 (JY654, Jinyuan, China) and human umbilical vein endothelial cells (HUVECs, ECV304, Bena, China) were utilized to assess the biocompatibility. The CCK-8 and Calcein/PI Assay Kit (C2015, Beyotime, China) were used for quantitative detection and visualization, respectively. In addition, the adhesion of MC3T3-E1 cells on the surface of the microspheres was assessed by staining the cells with DAPI.

### *In vitro* antibacterial performance

To investigate the antibacterial efficacy of IBPR-CuTA, *Staphylococcus aureus (S.aureus)* and *Escherichia coli (E.coli)* were selected. Sterilized IBP, IBPR and IBPR-CuTA microspheres were incubated with 100 μL bacterial suspension (1 × 10^5^ CFU/mL). The bacterial suspensions were plated onto agar plates. 24 h later, the number and morphology of bacterial colonies in each group were observed and photographed. To further assess bacterial viability following co-culture, a bacterial live/dead staining kit (SYTO9/PI; L7012, Thermo Fisher Scientific, USA) was used. Stained bacterial suspensions were imaged using a confocal laser scanning microscope (CLSM; DMi8, Leica, Germany). In addition, bacterial morphology was examined by scanning electron microscope (SEM; Gemini 300, ZEISS, Germany).

Furthermore, to evaluate the long-term antibacterial efficacy of IBPR-CuTA, *S.aureus* and *E.coli* at a concentration of 1 × 10^8^ CFU/mL were co-cultured with the samples. At predetermined time points, bacterial suspension was collected to calculate bacterial survival rates. Fresh bacterial suspensions were subsequently added to each sample to simulate prolonged bacterial challenge.

### Immunomodulatory response of macrophages

#### Intracellular ROS scavenging activity

To determine the capacity of intracellular ROS scavenging, a DCFH-DA ROS assay kit (S0033S, Beyotime, China) was employed. Briefly, RAW264.7 (CL-0190, Pricella, China) was stimulated with lipopolysaccharide (LPS; S11060, YuanYe, China). The cells were then co-cultured with sterilized IBP, IBPR, or IBPR-CuTA microspheres. After 24 h, intracellular ROS levels were examined using a fluorescence microscope and quantitatively detected by flow cytometry (Cytoflex, Beckman, USA).

#### Macrophage M1/M2 polarization

Macrophage M1/M2 polarization phenotypes were detected by immunofluorescence staining. After co-cultivation of RAW 264.7 cells with various materials for 2 days, anti-CD86 polyclonal antibody (1:500; 13395-1-AP, Proteintech, China) and anti-CD206/MRC1 mouse monoclonal antibody (1:500; AG2664, Beyotime, China) were used to detect macrophage polarization. Fluorescence images were captured using a CLSM. ImageJ software was employed for image analysis. In addition, the relative gene expression levels of tumor necrosis factor-alpha (*Tnf-α*), interleukin-1 beta (*Il-1β*), transforming growth factor-beta (*Tgf-β*) and interleukin-10 (*Il-10*) were analyzed by quantitative Real Time Polymerase Chain Reaction (qRT-PCR). Primer sequences are listed in Supplementary Table S1.

#### Transcriptome sequencing

Following the co-culture of RAW 264.7 macrophages with IBPR-CuTA for 2 days, samples were collected for RNA sequencing, with the LPS group serving as the control. Following quality evaluation, the libraries were prepared and then analyzed using high-throughput sequencing on the Illumina system (Oebiotech, China). Differential gene expression analysis between groups (IBPR-CuTA vs. Control) was performed using DESeq2. Genes with *P-*value < 0.05 and an absolute log2-fold change (|log2 FC|) > 0.58 were considered differentially expressed genes (DEGs). These DEGs were then subjected to enrichment analyses.

### *In vitro* evaluation of angiogenic activity

#### Scratch assay and tube formation assays

HUVECs were cultured to full confluence, and a linear scratch was made. After 12 and 24 h of incubation, cell migration across the scratched area was observed. The scratch width and the migration rate were quantitatively analyzed.

The angiogenic potential was further assessed via tube formation assays. Briefly, HUVECs were seeded onto wells pre-coated with Matrigel (356234, Corning, USA). Cell culture media containing the extracts of various materials were subsequently added. Tubular structures and tube formation parameters were quantitatively evaluated with the aid of ImageJ software.

#### Expression of CD31 and angiogenic genes

After co-culture of HUVECs with the different materials, samples were collected and subjected to immunofluorescence staining with a CD31 polyclonal antibody (1:250; 11265-1-AP, Proteintech, China). Fluorescence images were captured using CLSM, and relative fluorescence intensities were analyzed and normalized.

In addition, angiogenesis-related gene expression, including vascular endothelial growth factor (*VEGF*), endothelial nitric oxide synthase (*eNOS*) and angiopoietin-1 (*ANG-1*), was assessed by qRT-PCR after a 3-d co-culture. Primer sequences are listed in Supplementary Table S1.

### *In vitro* osteogenic activity evaluation

#### ALP and ARS staining

BMSCs isolated from rat femurs were co-cultured with IBP, IBPR, or IBPR-CuTA for 3 days and subsequently cultured in an osteogenic induction medium to induce osteogenic differentiation and assess their osteogenic potential. Alkaline phosphatase (ALP) activity was determined by BCIP/NBT ALP Kit (C3206, Beyotime, China) and ALP activity assay kit (BL862B, Biosharp, China). For mineralization assessment, a 2% Alizarin Red S (ARS) solution (C0138, Beyotime, China) was used.

#### Immunofluorescence staining

Cell spreading and adhesion are crucial factors affecting osteogenic differentiation. Therefore, the expression of F-actin and vinculin in BMSCs was assessed. After co-culture with the different materials, BMSCs were stained with an anti-vinculin antibody (1:200; ab129002, Abcam, UK) to label focal adhesions, while F-actin and nuclei were also stained. Fluorescence images were acquired using CLSM, and relative fluorescence intensities were normalized.

#### Expression of osteogenesis-related genes

The expression of osteogenic marker genes, including *Alp*, bone morphogenetic protein-2 (*Bmp-2*), runt-related transcription factor 2 (*Runx2*), collagen type I alpha 1 chain (*Col1a1*), osteopontin (*Opn*) and osteocalcin (*Ocn*), was analyzed by qRT-PCR. Primer sequences are listed in Supplementary Table S1.

### *In vivo* evaluation of infected bone defect

In this study, all animal experiments were approved by the Medical Ethics Committee of Southwest Jiaotong University (SWJTU-2103-024). Infected bone defects were constructed as previously described [39]. Briefly, 24 Sprague-Dawley rats (6 weeks, male) were randomly divided into four groups: control, IBP, IBPR and IBPR-CuTA. After acclimatization, a distal femoral bone defect (1 mm) was created in rats and infected with *S.aureus* for 1 week. Following debridement, the defect was filled with the experimental implant (control defects left unfilled), and rats were euthanized at 2 and 4 weeks post-implantation, and their femoral tissues were harvested for analysis. Additionally, at 2 weeks post-surgery, the defect site and surrounding tissues were collected for bacterial plating assays.

Micro-computed tomography (Micro-CT; VNC-102, PingSeng Healthcare, China) was performed at 50 kV and 200 μA to evaluate bone regeneration. The acquired scans were reconstructed and analyzed using Avatar3 software to obtain bone structural parameters.

Immunofluorescence staining was performed using macrophage markers CD86 (1:500; 30691-1-AP Proteintech, China) and CD206 (1:2000; 18704-1-AP, Proteintech, China). Immunohistochemical staining was used to evaluate angiogenesis and osteogenic markers, including VEGF (1:200; 26157-1-AP, Proteintech, China), OPN (1:200; 30200-1-AP, Proteintech, China) and OCN (1:200; 23418-1-AP, Proteintech, China), in the tissue sections.

### Statistical analysis

All quantitative experiments were performed with at least three independent biological replicates (*n* ≥ 3), and data are presented as mean ± standard deviation. Specific *n* values for each experiment are indicated in the figure legends. Statistical analyses were conducted using GraphPad Prism 9.5 software. Unpaired two-tailed Student’s *t*-tests were used for comparisons between two groups. For comparisons involving three or more groups, one-way analysis of variance (ANOVA) was employed, followed by Tukey’s honestly significant difference post-hoc test for multiple comparisons. *P < 0.05, **P < 0.01, ***P < 0.001, ****P < 0.0001, *ns*: no significant difference.

## Results and discussion

### Structural construction and physicochemical profiling of compartmentalized microspheres

BCP microspheres were fabricated by preparing a CaP precursor via the hydrothermal method, followed by high-temperature sintering. The surface of these BCP microspheres was covered with interconnected porous, wrinkled micro/nano-structures, giving them an overall appearance of coral-like microspheres (Supplementary Figure S1A). Compared with single-scale microspheres, calcium phosphate particles with a multilevel morphology can efficiently load drugs, adsorb protein molecules and promote osteogenesis [36, 40]. X-ray diffraction (XRD) patterns demonstrated the biphasic composition of BCP microspheres, with diffraction peaks at 25.9°, 31.8°, 32.2° and 32.9° correspond to the (002), (211), (112) and (300) crystal planes of hydroxyapatite (HA; JCPDS 09-0432), while peaks at 27.8°, 31.0° and 34.4° correspond to β-tricalcium phosphate (β-TCP; JCPDS 09-0169) (Supplementary Figure S1B). Compared with single-phase HA or TCP, BCP offers a superior balance between stability and resorbability, enabling more controlled degradation and better coupling between resorption and new bone formation [41]. As shown in Supplementary Figure S1C and D, these BCP microparticles exhibited a high specific surface area and porous structure (BET specific surface area S_BET_: 6.7074 m^2^/g, cumulative pore volume: 0.048862 cm³/g). This high porosity facilitates ICA loading and may promote cell infiltration and nutrient exchange during osteogenesis by providing abundant surface area, thereby contributing to new tissue formation [42]. Subsequently, these BCP microspheres were used to load the natural herbal compound ICA to enhance osteogenic activity. On the surface of these ICA@BCP microspheres, the attachment of ICA was observed and particle size analysis showed an average diameter of 4.66 ± 1.19 μm (Supplementary Figure S2).

**Figure 2:**
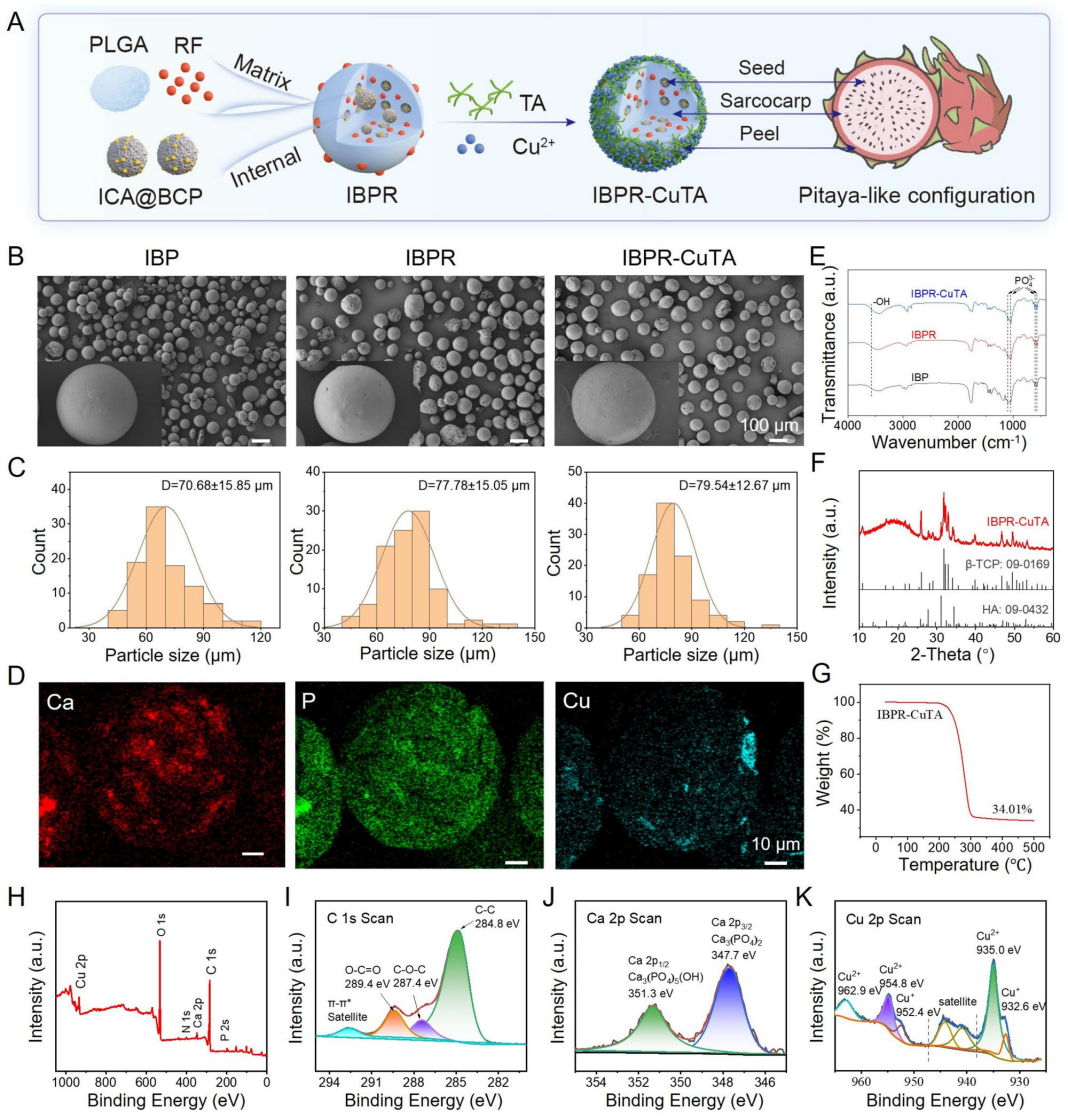
Structural construction and physicochemical profiling of pitaya‑inspired IBPR-CuTA microspheres. (**A**) Schematic illustration of the construction of pitaya-inspired IBPR‑CuTA microspheres with a compartmentalized architecture. (**B**) SEM images of IBP, IBPR and IBPR-CuTA. (**C**) Corresponding particle size distributions. (**D**) Elemental mapping of Ca, P and Cu in IBPR-CuTA. (**E**) FTIR spectra of IBP, IBPR and IBPR-CuTA. (**F**) XRD pattern of IBPR-CuTA. (**G**) TG curve of IBPR-CuTA. (**H-K**) XPS spectrum (H) and C 1s (I), Ca 2p (J), Cu 2p (K) scan elemental valence states of IBPR-CuTA.

Inspired by the configuration of pitaya, we developed compartmentalized IBPR-CuTA microspheres by embedding ICA@BCP particles (seed-like compartments) within an RF-loaded PLGA matrix (sarcocarp-like phase), followed by forming a surface CuTA metal coordination network (peel-like layer) (Figure 2A). IBP, IBPR and IBPR-CuTA microspheres all exhibited a regular spherical morphology, with IBPR-CuTA showing an outer CuTA coating attached to its surface (Figure 2B). Correspondingly, the mean microsphere diameter increased at each modification stage (drug loading and coating), as shown in Figure 2C. Energy-dispersive X-ray spectroscopy (EDS) elemental mapping confirmed a homogeneous distribution of Ca, P and Cu throughout the microspheres (Figure 2D; Supplementary Figure S3). Cross-sectional SEM images, coupled with EDS mapping, further demonstrated that the ICA@BCP component was uniformly dispersed throughout the microspheres in the form of multiple discrete internal compartments, with negligible agglomeration (Supplementary Figure S4). This compartmentalized internal architecture provides a structural basis for the spatiotemporally sequential release of the microspheres. Furthermore, the specific surface area and porosity of these microspheres were determined by BET analysis (Supplementary Figure S5). The results reveal a significant reduction in both the specific surface area (0.9828 m^2^/g) and total pore volume (0.10600 cm³/g) for the IBPR-CuTA microspheres. This can be primarily attributed to the encapsulation of the ICA@BCP core within a dense PLGA matrix, followed by the subsequent coating with the CuTA network. Such a relatively compact structure is crucial for preventing the burst release of encapsulated drugs and achieving sustained, controlled release [43]. This characteristic is highly beneficial for optimizing drug release kinetics, which is essential for long-term infection inhibition, effective immunomodulation and continuous support for bone regeneration.

Fourier transform infrared (FTIR) spectra showed that all sample groups exhibited characteristic absorption bands of PO_4_³^-^ at 1095, 1043, 603 and 567 cm^−1^, along with a distinct –OH stretching peak at 3571 cm^−1^. Additionally, the characteristic absorption bands of PLGA appeared in the 1200–2000 cm^−1^ range (Figure 2E). Notably, the XRD pattern of IBPR-CuTA retained characteristic peaks of BCP, indicating that the compartmentalized microsphere fabrication process did not disrupt the BCP crystalline structure (Figure 2F). Thermogravimetric (TG) analysis further showed that IBPR-CuTA had a residual mass of 34.01% at 500°C (Figure 2G). This inorganic fraction provides a mineral phase comparable to bone-like components, which contribute to structural stability, buffering of PLGA-derived acidity and osteogenic performance [29, 44]. X-ray photoelectron spectroscopy (XPS) revealed the presence of Cu 2p, O 1s, N 1s, Ca 2p and P 2s signals in the IBPR-CuTA microspheres. The appearance of an N 1s peak verified the successful loading of RF into IBPR-CuTA (Figure 2H). The π-π* resonance peak at 294–291 eV in the C 1s spectrum confirmed the incorporation of ICA (Figure 2I). Deconvolution of the Ca 2p spectrum showed peaks at 347.1 eV and 350.7 eV, corresponding to Ca_5_(PO_4_)_3_OH (HA) and Ca_3_(PO_4_)_2_ (β-TCP), confirming the biphasic composition of BCP (Figure 2J). Moreover, the high-resolution Cu 2p spectrum demonstrated a mixed Cu^2+^/Cu^+^ valence state (Figure 2K). This is likely attributable to the redox activity of the catechol moieties in TA, which can partially reduce Cu^2+^ to Cu^+^, thereby resulting in mixed-valence copper species. Such mixed-valence copper states are expected to be beneficial for antibacterial activity and redox cycling between Cu^2+^/Cu^+^ coupled with intracellular thiols may further amplify oxidative stress, membrane disruption and cytoplasmic leakage, particularly improving efficacy against Gram-positive bacteria [45–47]. As shown in Supplementary Figure S6, the zeta potential of different microspheres was evaluated. IBPR-CuTA microspheres had a zeta potential of about +1.78 mV, primarily due to the phenolic hydroxyl groups in their outer CuTA network. This positive surface charge typically promotes cell adhesion via enhanced electrostatic interactions with negatively charged cell membranes, which is beneficial for cell-material interactions and ultimately tissue regeneration [48].

### Injectability, pH-responsive drug release and antioxidant microenvironment modulation of IBPR-CuTA microspheres

**Figure 3:**
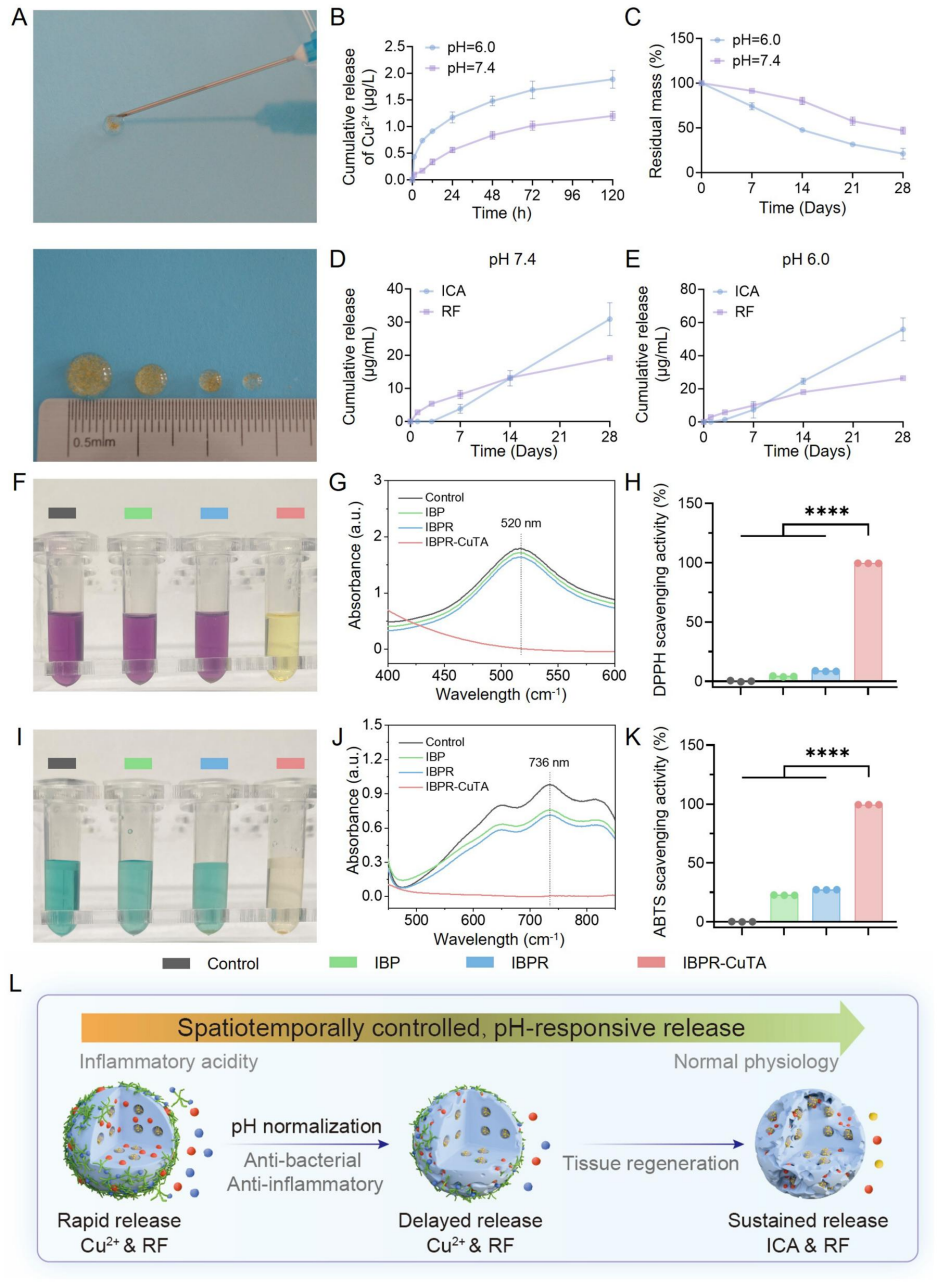
Injectability, pH-responsiveness and antioxidant activity of IBPR-CuTA. (**A**) Photograph of IBPR-CuTA extruded from a 23-gauge needle. (**B**) Cu^2+^ release profile of IBP-CuTA in PBS over 120 h. (**C**) Residual mass of IBP-CuTA during degradation in PBS over 28 days. Drug release profile of IBPR-CuTA in PBS at pH 7.4 (**D**) and pH 6.0 (**E**) over 28 days. (**F**) Photographs of DPPH solutions incubated with different groups. (**G**) Absorbance spectra of DPPH solutions after incubation with various groups in the range of 400–600 cm^−1^. (**H**) Quantitative analysis of DPPH radical scavenging activity. (**I**) Photographs of ABTS solutions incubated with different groups. (**J**) Absorbance spectra of ABTS solutions after incubation with various groups in the range of 450–850 cm^−1^. (**K**) Quantitative analysis of ABTS radical scavenging activity. (**L**) Schematic illustration of the pH‑responsive IBPR‑CuTA microspheres with sequential release capability for the programmed delivery of antibacterial, antioxidant and osteoinductive agents. *P < 0.05, **P < 0.01, ***P < 0.001, ****P < 0.0001.

Extrusion testing demonstrated that IBPR-CuTA microspheres could be smoothly injected through a 23-gauge needle, maintaining their structural integrity and dispersing uniformly (Figure 3A). The extrusion rate was calculated as the ratio of the extruded mass to the initially loaded mass. All groups exhibited extrusion rates above 95%, with no statistically significant differences after drug loading and coating (Supplementary Figure S7A). In addition, injection force is a key parameter for assessing implant injectability, and the commonly accepted maximum force is typically set at 10 N [49]. The average injection force required to extrude IBPR‑CuTA microspheres was 4.75 N, meeting the extrusion requirement for injectable composites (Supplementary Figure S7B). Collectively, these results highlight the excellent injectability of IBPR‑CuTA, enabling it to conformally fill irregular defect sites. Such an injectable microsphere is particularly advantageous for minimally invasive management of complex bone infections.

To achieve spatiotemporally controlled release in the drug-delivery platform, a pitaya-inspired compartmentalized configuration was designed to enable spatiotemporally controlled release and pH-responsive diffusion in IBPR-CuTA microspheres. To investigate the degradation behavior and sequential drug release profile, IBPR-CuTA microspheres were immersed in PBS under different pH conditions. The cumulative Cu^2+^ release curves revealed that the release rate of Cu^2+^ at pH 6.0 was significantly higher than at pH 7.4. In particular, at pH 6.0, a pronounced burst release of Cu^2+^ was observed within the first 12 h, followed by a slower but sustained release up to 120 h. By contrast, at pH 7.4, Cu^2+^ was released gradually and steadily, with no initial burst (Figure 3B). This pH-responsive release behavior indicated that in an acidic inflammatory environment (such as that caused by bacterial infection), IBPR-CuTA could rapidly release a high concentration of Cu^2+^ in the early hours, thereby synergizing with RF to inhibit pathogen growth. Under neutral physiological conditions associated with the later repair phase, however, only a low concentration of Cu^2+^ was released, which could aid in sustaining a pro-healing microenvironment by promoting angiogenesis while simultaneously supporting bone regeneration through stimulation of osteogenic differentiation and matrix mineralization, without excessive ion exposure that could compromise cytocompatibility [50, 51].

In addition, IBPR-CuTA exhibits degradation behavior that is responsive to pH. After 28 days of incubation, only 21.19% of the microsphere mass remained at pH 6.0, compared to 46.92% at pH 7.4 (Figure 3C), indicating faster degradation under acidic conditions. Drug release studies revealed a rapid release of RF within the first 3 days, which could provide early-stage antibacterial activity at the infection site. In contrast, ICA showed negligible release during this period because the ICA@BCP particles were embedded within the microspheres, and the PLGA matrix delayed the release process. After approximately 3 days, the gradual degradation of the material led to a sustained, near-linear release of both ICA and RF (Figure 3D and E; Supplementary Figure S8), thereby ensuring a long-term, steady supply of both agents during tissue repair [52]. The prolonged release of RF would help eliminate the risk of recurrent infection in the bone defect, while the sustained release of ICA was conducive to bone tissue regeneration by creating a pro-healing microenvironment [53].

To elucidate the dynamic release mechanism of these compartmentalized microspheres, the morphological changes of IBPR-CuTA microspheres during degradation and release under different pH conditions were characterized by SEM (Supplementary Figure S9A). In the initial stage, the outermost layer of the microspheres exhibited wrinkling and gradual degradation, indicating the preliminary breakdown of both the superficial CuTA network and the microsphere matrix. From Day 3 onwards, the PLGA shell degraded, exposing the internal ICA@BCP core. This dynamic degradation behavior continued with increasing degradation time and was closely associated with the pH environment. This observation is consistent with the drug release profiles exhibited by the microspheres, signifying the temporally controlled release effect achieved by the pitaya-inspired compartmentalized structure. Furthermore, analysis of the initial degradation process under varying pH environments revealed a reduction in Cu^2+^ binding energy and a shift in hydrogen bonds in the FTIR spectra (Supplementary Figure S9B–D), indicating weakened key chelating and hydrogen bonding interactions within the CuTA network [54]. These results support the presence of a compartmentalized architecture and suggest that this spatial organization may contribute to the observed pH-responsive and temporally regulated release behavior.

Antioxidant activity facilitates tissue regeneration through multiple synergistic mechanisms, including maintenance of redox homeostasis, modulation of the immune microenvironment and activation of repair-associated pathways. These combined effects make antioxidant approaches a key strategy in tissue engineering [55]. We evaluated the radical scavenging activities of IBPR-CuTA against DPPH and ABTS assays. Compared with the other groups, IBPR-CuTA exhibited remarkably high radical scavenging capacities in both assays, reaching 99.51% and 99.39% in the DPPH and ABTS assays, respectively. In contrast, the IBP and IBPR groups, as well as the control, showed only negligible scavenging activity (Figure 3F–K). This exceptional antioxidant performance was attributed to the abundant phenolic hydroxyl groups present in the TA coating of IBPR-CuTA, which neutralized free radicals by donating hydrogen atoms or electrons [56]. These results suggests that IBPR-CuTA held great promise for regulating the inflammatory microenvironment in infected bone defects.

In summary, the pH‑responsive IBPR‑CuTA microspheres with sequential release capability enable the programmed delivery of antibacterial, antioxidant and osteoinductive agents, thereby offering a promising strategy to address the complex microenvironment of infectious bone defects (Figure 3L).

### *In vitro* biocompatibility and antibacterial activity of IBPR-CuTA

**Figure 4:**
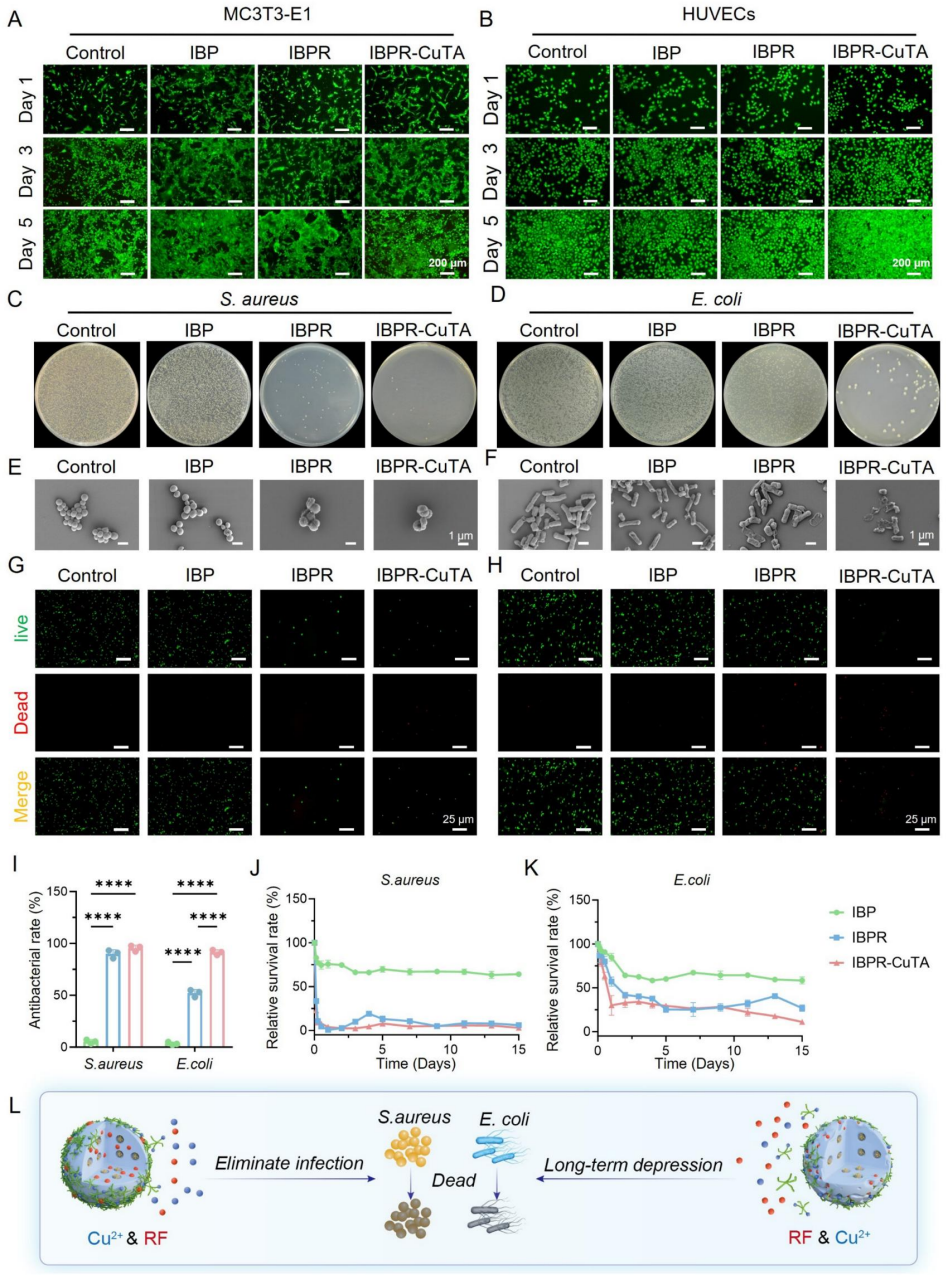
*In vitro* biocompatibility and antibacterial evaluation of IBPR-CuTA. Live/dead staining images of (**A**) MC3T3-E1 and (**B**) HUVECs were co-cultured with different groups for 1, 3 and 5 days. Images of (**C**) *S.aureus* and (**D**) *E.coli* colonies on agar plates after 24 h of culture. SEM images showing the morphology of (**E**) *S.aureus* and (**F**) *E.coli*. Live/dead staining images of (**G**) *S.aureus* and (**H**) *E.coli*. (**I**) Antibacterial rate after 24 h. Bacterial survival rates of (**J**) *S.aureus* and (**K**) *E.coli* after 15 days of co-culture with different groups. (**L**) Schematic illustration of the dual antibacterial strategy of IBPR‑CuTA microspheres, featuring rapid infection elimination and long‑term depression. *P < 0.05, **P < 0.01, ***P < 0.001, ****P < 0.0001.

Biomaterials intended for clinical implantation are generally expected to maintain excellent biocompatibility while also exhibiting potent antibacterial activity [57]. Establishing an outstanding biocompatibility profile and robust antibacterial performance was crucial for validating IBPR‑CuTA as a safe and effective implant platform for bone tissue repair and infection control. Hemolysis assays showed that all material groups had hemolysis rates below 5%, indicating no hemolytic reaction and thus good blood compatibility (Supplementary Figure S10). Live/dead cell staining revealed that none of the tested materials adversely affected the proliferation of MC3T3-E1 osteoblasts or HUVECs, with negligible cell death observed (Figure 4A and B). Moreover, CCK-8 assays demonstrated that IBPR-CuTA significantly promoted the proliferation of both cell types compared to the other groups (Supplementary Figure S11), likely due to the introduction of the CuTA coating, which can effectively stimulate cell growth. Taken together, these results demonstrate that IBPR-CuTA possesses excellent biocompatibility, establishing a foundation for its use in tissue repair applications. Cell adhesion to biomaterials is fundamental for regulating cell fate, including proliferation, differentiation and migration [58]. In this study, we used MC3T3‑E1 cells to assess the cell adhesion capacity of different microsphere surfaces. Compared with the IBP and IBPR groups, the IBPR‑CuTA surfaces exhibited a greater number of cell nuclei, indicating superior cell adhesion capacity (Supplementary Figure S12). These results suggest that the CuTA complex coating effectively improved the cell-adhesive properties of the compartmentalized microspheres. By modulating adhesion properties, IBPR‑CuTA is expected to reshape the local microenvironment, thereby influencing the behavior of osteoblasts and immune cells [59].

Given the pH-responsive and sequential release behavior of IBPR‑CuTA, which enables rapid Cu^2+^ release under acidic conditions followed by sustained RF release (Figure 3B–E), this drug-delivery platform is expected to provide broad-spectrum bactericidal activity and long-term inhibitory effects against representative pathogens [60, 61]. *Staphylococcus aureus* (Gram-positive) and *E.coli* (Gram-negative) represent two of the primary bacteria responsible for bone infections, with *S.aureus* accounting for approximately 31.4% of cases [62]. Plate-counting experiments showed that samples containing RF (IBPR and IBPR-CuTA) significantly inhibited *S.aureus* colony formation compared to the control and IBP groups (Figure 4C), with IBPR-CuTA exhibiting the strongest antibacterial effect. In cultures of *E.coli*, some colonies were still observed in the IBPR group, whereas the IBPR-CuTA group showed a more pronounced inhibitory effect (Figure 4D). SEM imaging further revealed morphological changes in bacteria following treatment with IBPR-CuTA. In the control group, *S.aureus* exhibited a typical smooth, spherical morphology and *E.coli* maintained a rod-like form with intact cell surfaces. In contrast, both bacteria in the IBPR-CuTA group displayed significant deformation, including cell shrinkage and membrane rupture (Figure 4E and F). Live/Dead fluorescence staining provided additional evidence of the antibacterial efficacy of IBPR-CuTA. In control groups, images exhibited strong green fluorescence, indicating high bacterial viability, whereas the IBPR and IBPR-CuTA groups showed markedly diminished green fluorescence (Figure 4G and H). This indicated that both RF-loaded samples had significant bactericidal effects, with IBPR-CuTA being particularly effective. Quantitative antibacterial assays based on optical density measurements further corroborated these findings. Both IBPR and IBPR-CuTA exhibited high antibacterial efficacy, with IBPR-CuTA achieving bactericidal rates exceeding 90% in both *S.aureus* and *E.coli* (Figure 4I). Given the critical role of bacterial biofilm formation in persistent bone infections, we evaluated the ability of IBPR-CuTA microspheres to inhibit *S.aureus* and *E.coli* biofilm formation using the crystal violet staining method, as reported in the literature [63]. As shown in Supplementary Figure S13A, the IBPR group, loaded with RF only, exhibited limited antibiofilm capability. In contrast, IBPR-CuTA significantly inhibited the formation of both *S.aureus* and *E.coli* biofilms. Quantitative analysis revealed that IBPR-CuTA demonstrated the strongest antibiofilm effect against both strains, leading to a substantial decrease in absorbance values and almost complete eradication of biofilm formation (Supplementary Figure S13B and C). These results confirm that IBPR-CuTA possesses potent, broad-spectrum antibiofilm capabilities, offering significant advantages for treating complex infected bone defects where biofilm eradication is crucial. The enhanced antimicrobial performance may be associated with the combined contributions of RF and the CuTA coating. On one hand, CuTA controllably releases Cu^2+^ under the stimulation of the infected microenvironment. Once excessive Cu^2+^ enters bacterial cells, it triggers a cuproptosis-like effect, disrupting the tricarboxylic acid cycle and inhibiting energy metabolism [64, 65]. It can also enhance binding through electrostatic interactions, physically damage bacterial membranes, accelerate the leakage of cellular contents, lead to bacterial death and disrupt the biofilm matrix [66, 67]. On the other hand, as a rifamycin antibiotic, RF specifically binds to the β-subunit of bacterial RNA polymerase, thereby blocking mRNA synthesis and inhibiting bacterial protein synthesis and proliferation [68].

Because bone infection treatment often requires sustained antibacterial activity over extended periods [26], the long-term antibacterial performance of the materials was evaluated over 15 days. Both IBPR and IBPR-CuTA demonstrated prolonged antibacterial effects against *S.aureus*, with bacterial survival rates of 6.03% and 3.11%, respectively, whereas the IBPR-CuTA group exhibited the most pronounced long-term effect against *E.coli*, maintaining an inhibition rate above 90% at 15 days (Figure 4J and K). These results indicated that IBPR-CuTA employed a dual antibacterial strategy, rapidly eliminating pathogens within the first day through the release of RF and Cu^2+^ and sustaining long-term high-level antibacterial activity as the carrier degrades and continuously releases RF (Figure 4L).

### ROS regulation and macrophage polarization by IBPR-CuTA

**Figure 5:**
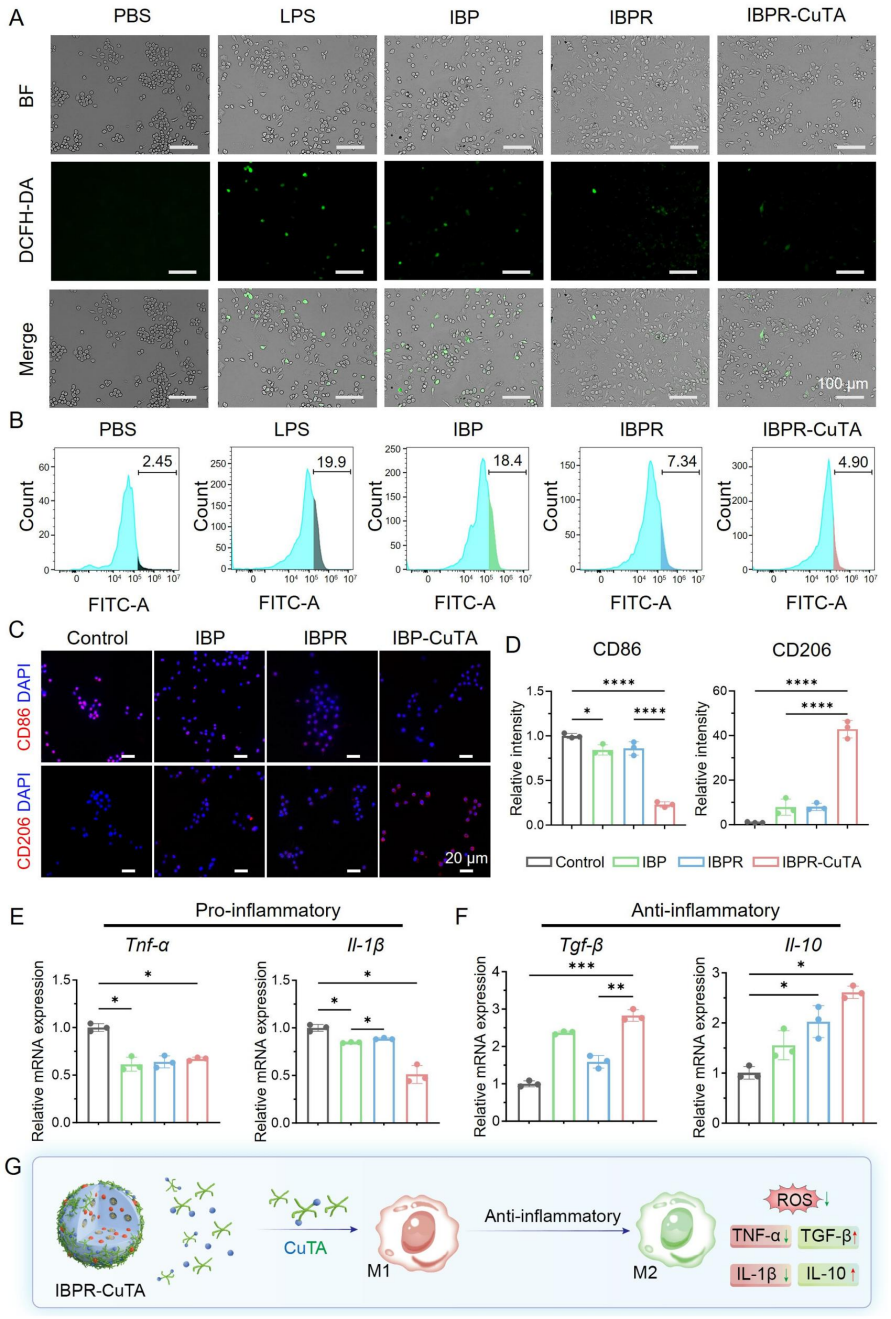
*In vitro* macrophage immune responses to IBPR-CuTA. (**A**) Fluorescence images showing intracellular ROS in RAW 264.7 cells. (**B**) Flow cytometric analysis of ROS levels in RAW 264.7 cells. (**C**) CLSM images showing expression of CD86 and CD206. (**D**) Quantification of relative fluorescence intensities of CD86 and CD206. (**E**) Relative mRNA expression levels of pro-inflammatory genes *Tnf-α* and *Il-1β*. (**F**) Relative mRNA expression levels of anti-inflammatory genes *Tgf-β* and *Il-10*. (**G**) Schematic illustration of the anti‑inflammatory effect of IBPR‑CuTA microspheres. *P < 0.05, **P < 0.01, ***P < 0.001, ****P < 0.0001.

Excessive accumulation of ROS is commonly observed in infected bone defect regions, where it disrupts the bone immune microenvironment, impairs osteogenic cell function and hinders bone regeneration [69, 70]. Eliminating ROS can reverse this pathological microenvironment and create favorable conditions for bone repair [71]. To evaluate the antioxidant performance of IBPR-CuTA, we measured intracellular ROS in LPS-stimulated RAW264.7 cells. LPS stimulation caused a pronounced increase in ROS levels, whereas all material-treated groups exhibited significantly lower ROS levels. The IBPR-CuTA group showed the weakest DCF fluorescence signal, indicating the most effective ROS scavenging (Figure 5A). Flow cytometry further corroborated these findings. In the LPS-only group, ∼19.9% of cells were ROS-positive, whereas IBPR and IBPR-CuTA treatments markedly reduced this fraction. The IBPR-CuTA group had only ∼4.9% ROS-positive cells, essentially returning to basal levels (Figure 5B). This pronounced antioxidant effect of IBPR-CuTA is primarily attributed to the phenolic hydroxyl groups in its CuTA coating, which confer intrinsic free-radical-scavenging properties. Moreover, CuTA mimics the activity of superoxide dismutase and catalase, thereby markedly diminishing intracellular ROS and protecting cells from oxidative stress-induced damage [72, 73].

Macrophage polarization plays an essential role in bone regeneration. Following biomaterial implantation, a timely shift of macrophages from the pro-inflammatory M1 phenotype to the anti-inflammatory M2 phenotype is critical for successful bone regeneration. To examine the influence of IBPR-CuTA on this process, we incubated RAW264.7 cells with IBP, IBPR or IBPR-CuTA. CLSM images and quantitative analysis revealed that the IBPR-CuTA group had markedly lower CD86 expression than the other groups, coupled with significantly higher CD206 expression (Figure 5C and D). These results indicated that IBPR-CuTA effectively inhibited M1 polarization while promoting macrophage transition to the M2 phenotype. Given that the balance between pro- and anti-inflammatory cytokines secreted by macrophages played a vital role in bone regeneration [74–76], we subsequently assessed the expression of key cytokine genes in RAW264.7 cells using qRT-PCR. All material-treated groups significantly downregulated the expression of *Tnf-α* and *Il-1β* relative to the control, with IBPR-CuTA eliciting the most pronounced suppression of *Il-1β* (Figure 5E). Conversely, IBPR-CuTA significantly upregulated *Tgf-β* and *Il-10*, resulting in levels significantly higher than those in the other groups (Figure 5F). Taken together, these results demonstrated that IBPR-CuTA profoundly modulated macrophage polarization and the inflammatory response, an effect primarily attributed to the release of TA and Cu^2+^ during the first 3 days (Figure 5G). This exceptional immunomodulatory capacity created a favorable immune microenvironment for bone defect repair, thereby further promoting bone tissue regeneration and remodeling [77].

### Mechanism of IBPR-CuTA in macrophage regulation

**Figure 6:**
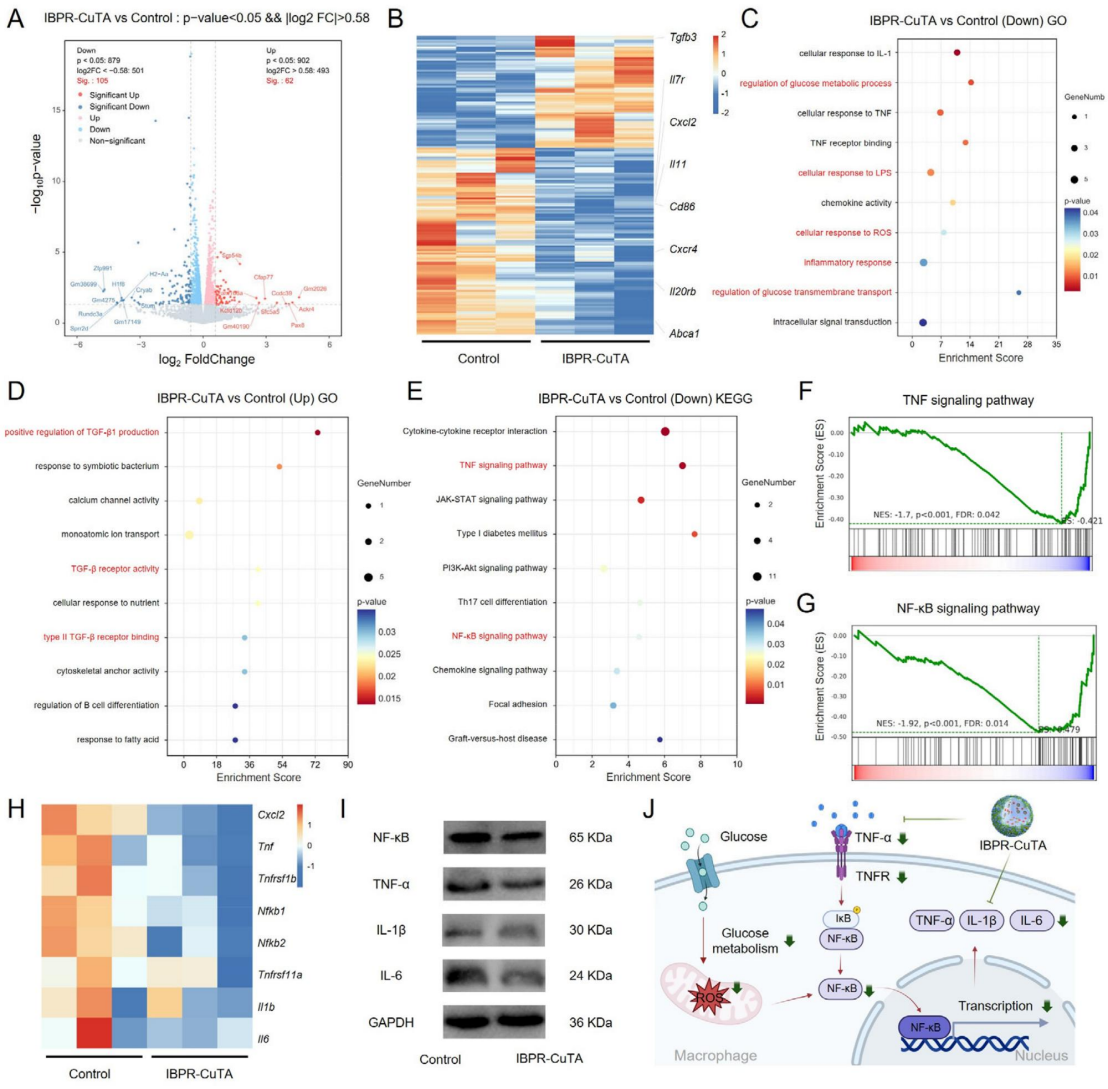
Mechanistic analysis of IBPR-CuTA-mediated immunomodulation in RAW 264.7 macrophages. (**A**) Volcano plot of DEGs between IBPR-CuTA and control groups. (**B**) Heatmap of DEGs between IBPR-CuTA and control groups. GO enrichment analysis of (**C**) downregulated and (**D**) upregulated DEGs. (**E**) KEGG enrichment analysis of downregulated DEGs. GSEA of (**F**) TNF and (**G**) NF-κB signaling pathways. (**H**) Heatmap of key gene expression in the TNF/NF-κB signaling pathway. (**I**) WB analysis showing protein expression levels of TNF-α, NF-κB, IL-1β and IL-6. (**J**) Schematic diagram of signaling pathways involved in IBPR-CuTA-induced macrophage polarization.

To further elucidate the mechanism by which IBPR-CuTA modulates macrophages, we performed transcriptome sequencing for in-depth analysis. The results showed that IBPR-CuTA significantly upregulated 62 and downregulated 102 genes, as identified by differential expression analysis (Figure 6A). Focusing on inflammation-related genes, IBPR-CuTA markedly downregulated several pro-inflammatory and chemokine genes (*Cd86*, *Tnf*, *Nos2*, *Nfkb2*, *Cxcl2*, *Cxcr4*) while upregulating anti-inflammatory genes (*Tgfb3*, *Tgfbr3l*, *Il10*, *Il10ra*) (Figure 6B; Supplementary Figure S14). These transcriptomic changes suggested a shift toward an anti-inflammatory gene expression profile in IBPR-CuTA-treated macrophages.

Gene Ontology (GO) enrichment analysis further corroborated the functional impact of IBPR-CuTA. The GO terms downregulated by IBPR-CuTA were predominantly related to inflammation and metabolism. Specifically, they included regulation of glucose metabolic process, cellular responses to LPS and ROS, the inflammatory response, glucose transmembrane transport and the production and activity regulation of multiple pro-inflammatory factors (Figure 6C). To validate the inhibitory effect of IBPR-CuTA on glucose metabolism, we utilized western blot (WB) analysis to detect the protein expression levels of Glucose transporter 1 (GLUT1), pyruvate kinase M2 (PKM2) and hexokinase 2 (HK2), all key components in glucose metabolism. The results demonstrated that IBPR-CuTA significantly downregulated the expression levels of these proteins, further supporting that IBPR-CuTA downregulates glucose metabolism-related proteins (Supplementary Figure S15). Glucose metabolism serves as a central hub controlling macrophage phenotype and function during tissue healing. By modulating metabolic pathways, the balance between pro-inflammatory (M1) and reparative (M2) macrophage polarization can be shifted, thereby transforming the microenvironment from a pro-inflammatory state to one conducive to inflammation resolution and new bone formation [78]. In line with this concept, strategies that inhibit macrophage glycolysis while enhancing mitochondrial oxidative metabolism have been shown to alleviate inflammation and promote bone formation [79]. Conversely, the genes upregulated by IBPR-CuTA were enriched in processes associated with TGF-β signaling. These processes included TGF-β production, regulation of TGF-β activity and receptor binding (Figure 6D). These results indicated that IBPR-CuTA effectively suppressed inflammatory responses and promoted tissue-repair processes, likely through metabolic reprogramming of macrophages. Consistent with these findings, Kyoto Encyclopedia of Genes and Genomes (KEGG) pathway analysis revealed that IBPR-CuTA significantly downregulated key inflammation-associated signaling pathways, particularly the TNF and NF-κB pathways (Figure 6E). Furthermore, gene set enrichment analysis (GSEA) confirmed that both the TNF and NF-κB signaling cascades were markedly inhibited by IBPR-CuTA (Figure 6F and G). The TNF signaling pathway, mediated by the TNF family (especially TNF-α), plays a pivotal role in inflammatory responses, immune regulation and cell differentiation and apoptosis [80, 81]. TNF-α binds to its cell-surface TNF receptor (TNFR) to activate the NF-κB pathway, thereby exerting its biological effects [82]. Subsequently, NF-κB translocates to the nucleus and induces the expression of numerous inflammatory genes, acting as a key regulator in the initiation, modulation and resolution of inflammation [83, 84]. At the gene level, IBPR-CuTA significantly downregulated multiple key mediators of the TNF/NF-κB axis. These included *Tnf* itself, several TNFR genes (e.g. *Tnfrsf1b* and *Tnfrsf11a*) and NF-κB subunit genes (e.g. *Nfkb1* an*d Nfkb2*). IBPR-CuTA also reduced the mRNA levels of other pro-inflammatory cytokines, such as *Il1b* and *Il6* (Figure 6H). Consistent with the transcriptomic data, WB analysis confirmed corresponding reductions at the protein level: IBPR-CuTA treatment decreased the expression of TNF-α, NF-κB, IL-1β and IL-6 proteins, in line with the mRNA findings (Figure 6I). Together, the observed suppression of the TNF and NF-κB inflammatory signaling pathways by IBPR-CuTA suggested that this treatment may exert its therapeutic effects by inhibiting macrophage glycolytic activity (M1) while promoting characteristics of reparative (M2) macrophages (Figure 6J). These actions markedly reduced the secretion of pro-inflammatory cytokines (e.g. TNF-α, IL-1β, IL-6), thereby fostering a friendly microenvironment for resolving inflammation and promoting bone tissue repair.

### *In vitro* angiogenic activity of IBPR-CuTA

**Figure 7:**
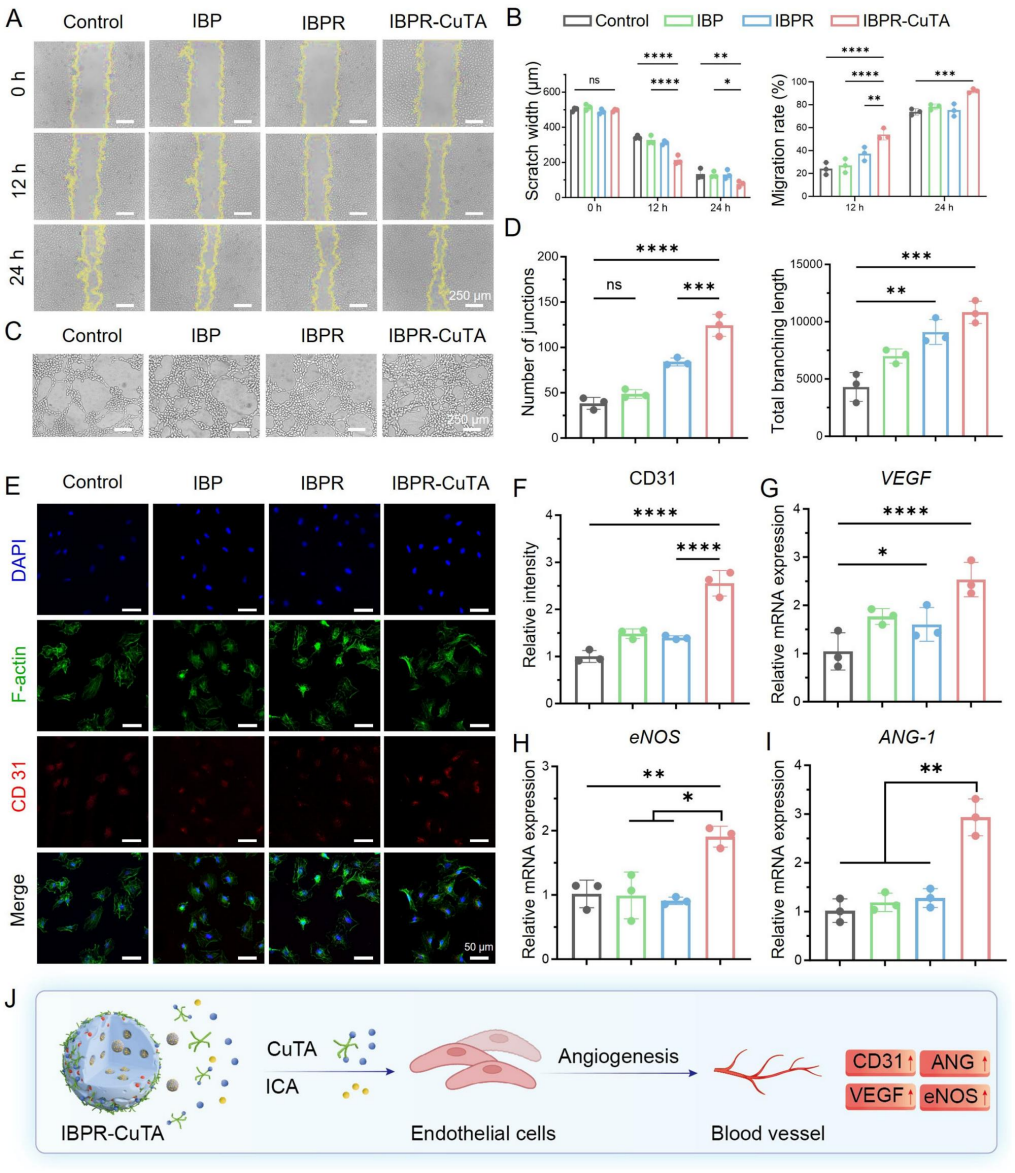
*In vitro* angiogenic activity of IBPR-CuTA. (**A**) Optical micrographs of the HUVECs scratch assay. (**B**) Quantification of the mean scratch width and migration rate. (**C**) Optical micrographs of HUVECs tube formation on Matrigel after 6 h. (**D**) Quantification of the number of junctions and total branching length. (**E**) CLSM images of CD31-immunostained HUVECs after 24 h of culture. (**F**) The relative fluorescence intensity of CD31. qRT-PCR analysis of the relative mRNA expression levels of (**G**) *VEGF*, (**H**) *eNOS* and (**I**) *ANG-1* in HUVECs after 3 days of culture. (**J**) Schematic illustration of the pro‑angiogenic activity of IBPR‑CuTA microspheres. *P < 0.05, **P < 0.01, ***P < 0.001, ****P < 0.0001, *ns*, not significant.

Infected bone defects, characterized by infection, inflammation and necrotic bone tissue that compromise local blood supply and impede healing, highlight the critical importance of angiogenesis in bone repair [85]. Enhancing vascular regeneration by improving nutrient delivery, modulating inflammation and recruiting bone repair cells accelerates bone healing and has become an essential component of bone regeneration and tissue engineering strategies [86, 87]. We therefore evaluated the *in vitro* angiogenic activity of IBPR-CuTA. A scratch assay with HUVECs was performed to assess endothelial cell migration. In the IBPR-CuTA group, the scratch was almost completely closed within 24 h, indicating markedly promoted HUVECs migration relative to the IBP and IBPR groups (Figure 7A). Quantification of the scratch width and the migration rate showed that the IBPR-CuTA group had a markedly narrower average scratch width and a larger migration rate (Figure 7B). These findings indicated that the Cu^2+^ released from the CuTA component effectively enhanced the promotion of endothelial cell migration by IBPR-CuTA, laying a foundation for subsequent pro-angiogenic cell proliferation and migration [88]. The role of IBPR-CuTA in angiogenesis was further examined using an HUVEC tube formation assay on Matrigel. The IBPR-CuTA group exhibited the most robust capillary-like network formation, with the highest number of tubular structures, significantly greater total tube length and a more continuous, highly branched network compared to all other groups (Figure 7C). Quantitative analysis confirmed that the number of junctions and total branching length were markedly enhanced in the IBPR-CuTA group, suggesting an enhanced ability to support neovascularization (Figure 7D).

To evaluate CD31 and cytoskeletal F-actin of endothelial cells, immunofluorescence staining was performed in HUVECs. CD31 is a key early marker of vascular development, and F-actin reorganization is closely related to endothelial cell migration, invasion and angiogenesis [89]. The IBPR-CuTA group displayed the strongest CD31 fluorescence signal, indicating pronounced pro-angiogenic characteristics (Figure 7E and F). Quantitative analysis of fluorescence intensity revealed that CD31 expression in the IBPR-CuTA group was significantly higher than in other groups (*P *< 0.0001), highlighting the superior efficacy of IBPR-CuTA in promoting the endothelial phenotype and facilitating vessel formation.

In addition, angiogenesis-related gene expression was analyzed by qRT-PCR. The expression of *VEGF, eNOS* and *ANG-1* in the IBP and IBPR groups showed no significant differences relative to control, whereas in the IBPR-CuTA, these genes were markedly upregulated (Figure 7G–I). The observed upregulation is inferred to be associated with the Cu^2+^ and ICA liberated from IBPR-CuTA (Figure 7J). We speculate that this liberated Cu^2+^ may initiate the HIF-1α signaling pathway, thereby contributing to the stimulation of endothelial cell function and the provision of essential molecular signals that promote angiogenesis [90–92]. Further studies are warranted to confirm this mechanistic link.

### *In vitro* osteogenic activity of IBPR-CuTA

**Figure 8:**
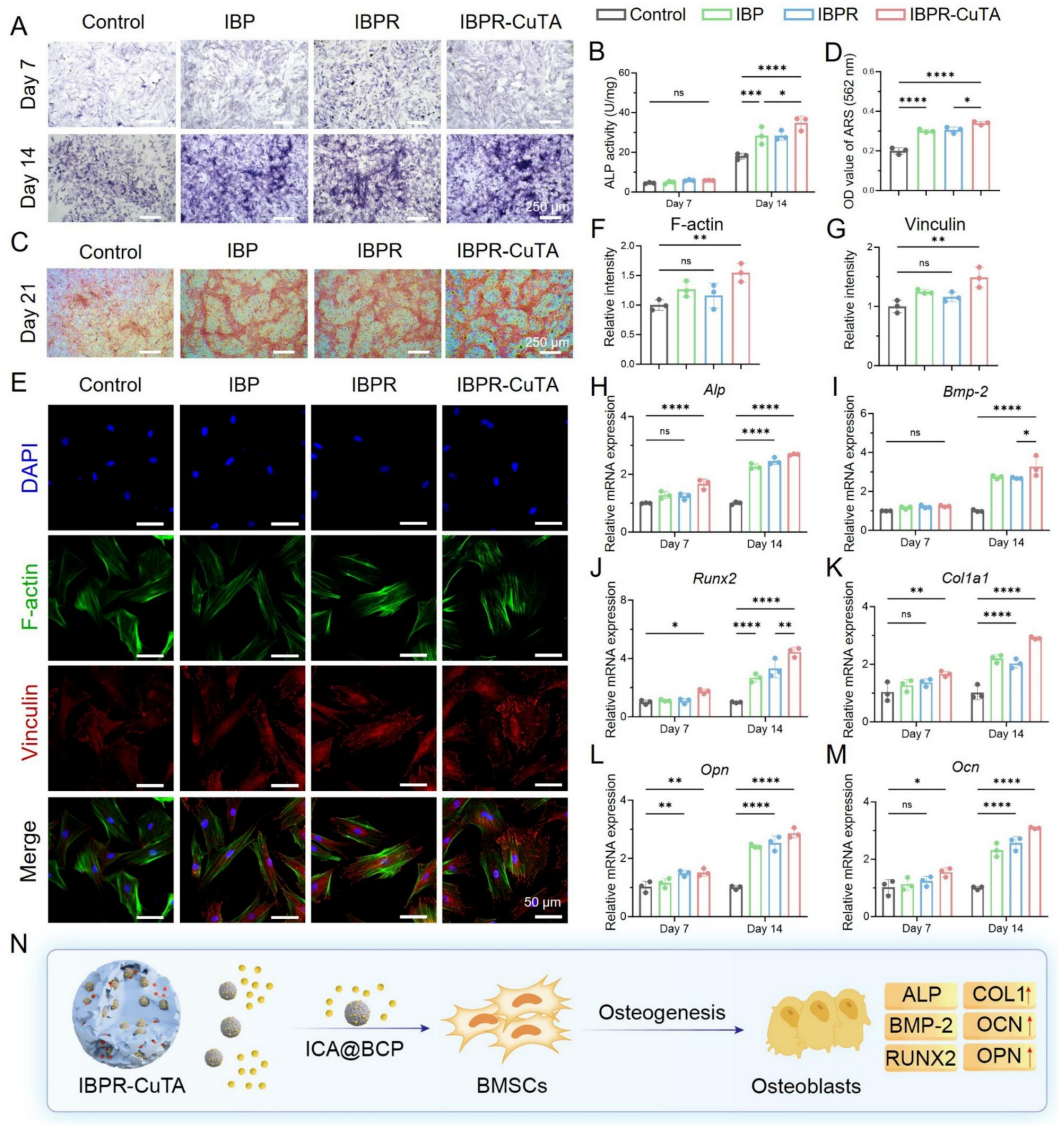
*In vitro* osteogenic activity of IBPR-CuTA. (**A**) ALP staining and (**B**) ALP activity for BMSCs co-cultured with the materials for 7 and 14 days. (**C**) ARS staining and (**D**) quantification for BMSCs co-cultured for 21 days. (**E-G**) CLSM images of immunofluorescent staining of the cytoskeleton (F-actin) and focal adhesion protein (vinculin) in BMSCs co-cultured for 3 days (**E**), with quantitative analysis of F-actin immunofluorescent intensity (**F**) and vinculin expression (**G**). qRT-PCR analysis of osteogenic gene expression of (**H**) *Alp*, (**I**) *Bmp-2*, (**J**) *Runx2*, (**K**) *Col1a1*, (**L**) *Opn* and (**M**) *Ocn*. (**N**) Schematic illustration of the osteogenic activity of IBPR‑CuTA microspheres. *P < 0.05, **P < 0.01, ***P < 0.001, ****P < 0.0001, *ns*, not significant.

BMSCs are among the primary cells involved in bone formation and play a crucial role in bone repair, with their osteogenic differentiation serving as an essential step in bone regeneration, thereby underscoring the need to evaluate the osteogenic effect of IBPR-CuTA. ALP expression reflects the extent of osteogenic activity and the initiation of functional differentiation in BMSCs [93]. ALP staining and activity assays revealed significantly higher ALP expression at Day 14 relative to Day 7 in all groups (Figure 8A and B). At 21 days, ARS staining and quantification demonstrated that the control group formed relatively few mineralized nodules, whereas all material-treated groups exhibited increased staining intensity. Notably, the IBPR-CuTA group showed the most pronounced mineralized nodule formation, with quantification indicating a significantly higher level of calcification than other groups (Figure 8C and D). These results indicated that IBPR-CuTA not only significantly promoted early osteogenic differentiation but also played an important role in the mineralization process. This phenomenon may result from the sustained release of ICA and BCP after Day 3, which stimulated osteogenic differentiation in BMSCs [94]. In addition, Cu^2+^ release from IBPR-CuTA further enhances osteogenic activity [73].

In osteogenic differentiation, the focal adhesion protein vinculin serves as a key regulatory molecule. High vinculin expression is typically associated with reinforced focal adhesions and cytoskeletal remodeling, which are early steps in osteogenesis. BMSCs interact with the extracellular environment via focal adhesions, initiating signaling cascades that drive the expression of osteogenesis-related genes (e.g. *Runx2*, *Alp*, *Ocn*), activate differentiation pathways and enhance osteogenic capacity [95]. BMSCs cultured with IBPR-CuTA exhibited the strongest vinculin fluorescence signal and the most well-developed cytoskeletal structure among all groups (Figure 8E), consistent with quantitative analyses of F-actin intensity and vinculin expression, which confirmed that the IBPR-CuTA group was significantly superior to the other groups (Figure 8F and G). Thus, IBPR-CuTA enhanced vinculin expression and improved cytoskeletal remodeling, providing a stronger cellular foundation for osteogenic differentiation.

The osteogenic gene expression was next determined through qRT-PCR. The results showed that IBPR-CuTA significantly upregulated several key osteogenic markers. In the IBPR-CuTA group, *Bmp-2*, a critical driver of osteogenic signaling, was significantly elevated (*P *< 0.001), and *Alp* and *Runx2*, early osteogenic markers and essential transcription factors, were expressed at markedly higher levels (*P *< 0.01), indicating activation of early osteogenic differentiation and reflecting the synergistic effects of ICA and Cu^2+^. In addition, IBPR-CuTA substantially increased *Col1a1* expression, suggesting that the combination of BCP with ICA is particularly effective during the bone matrix formation stage. Furthermore, the late-stage markers *Ocn* and *Opn* were significantly upregulated by Day 14 in the IBPR-CuTA group (*P *< 0.01), further suggesting the outstanding effect of IBPR-CuTA in promoting bone maturation and mineralization (Figure 8H–M). Taken together, these results indicated that from Days 7 to 14, the sustained and stable release of BCP and ICA from IBPR-CuTA significantly elevated the expression of crucial osteogenic genes. This enhanced effect spanned the entire differentiation process from early-stage differentiation (*Alp, Runx2*) to late-stage maturation and mineralization (*Ocn*, *Opn*), demonstrating a comprehensive promotion of osteogenesis and providing strong evidence for the potential of IBPR-CuTA in bone regeneration therapy. These results indicate that the IBPR‑CuTA microspheres achieve efficient osteogenic activity through the synergistic effects of ICA and BCP (Figure 8N). This osteogenic effect of IBPR-CuTA is hypothesized to involve activation of osteogenesis-related signaling pathways, such as BMP/SMAD-Runx2, and Wnt/β‑catenin, potentially along with Ca^2+^-dependent signaling and adhesion-mediated mechanotransduction [96].

### *In vivo* repairing infected bone defects of IBPR-CuTA

**Figure 9:**
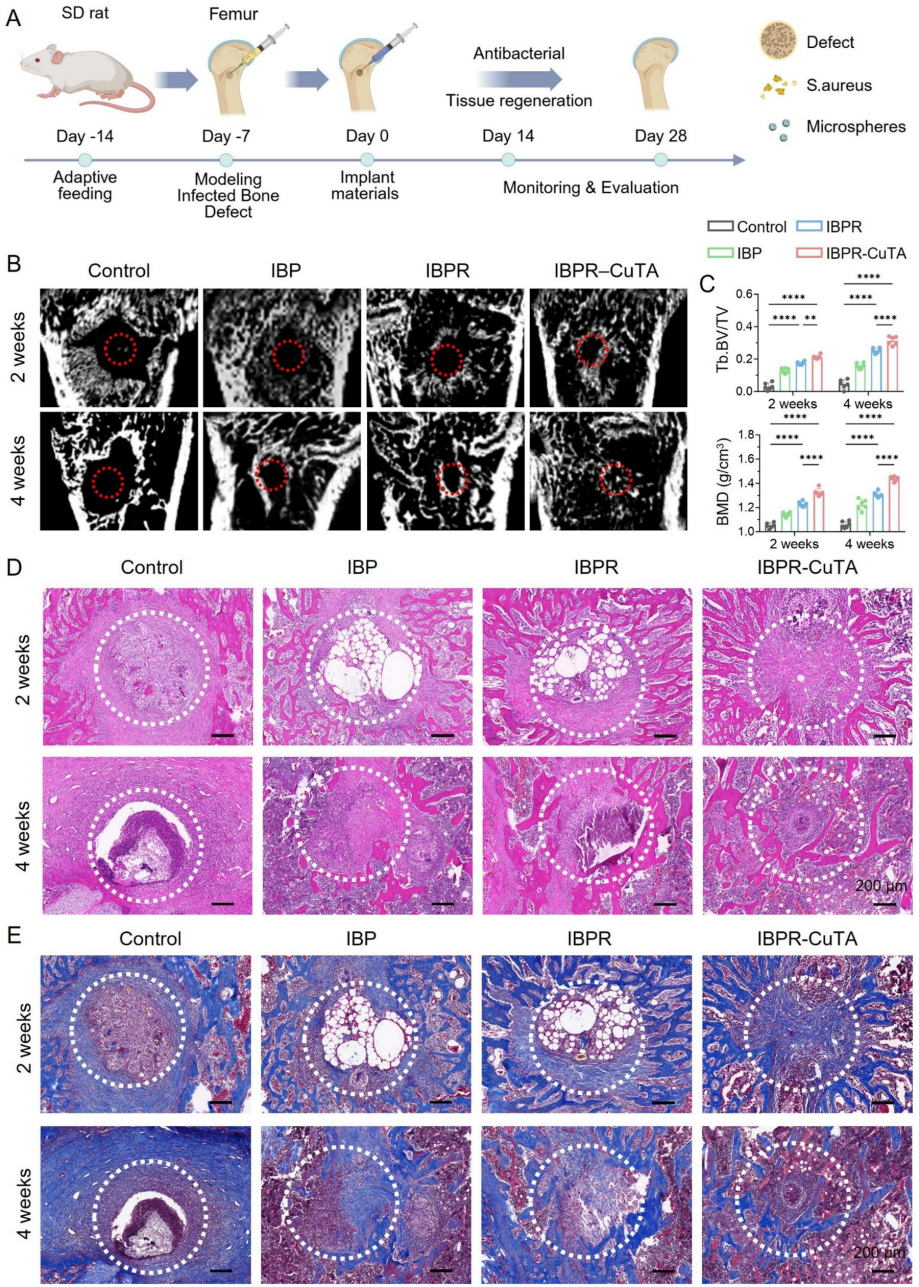
*In vivo* repair of infected bone defects using IBPR-CuTA. (**A**) Schematic illustration of the infected bone defect model establishment and IBPR-CuTA implant placement. (**B**) Micro-CT images at 2 and 4 weeks post-implantation. (**C**) Quantitative analysis of bone structural parameters (Tb.BV/TV, BMD). (**D**) H&E-stained images. (**E**) Masson’s trichrome-stained images. *P < 0.05, **P < 0.01, ***P < 0.001, ****P < 0.0001.

To evaluate the *in vivo* therapeutic efficacy of IBPR-CuTA, an *S.aureus*-infected bone defect model was established in rats by drilling a 1-mm hole in the distal femur and injecting a bacterial suspension (Figure 9A). After 7 days, infection was confirmed by positive *S.aureus* cultures and micro-CT imaging, which showed significant bone resorption and infection-induced bone destruction in the defect region (Supplementary Figure S16). The infected defects were then treated with different materials and assessed at 2 and 4 weeks following surgery. At 2 weeks, plate culture analysis showed that both IBPR and IBPR-CuTA markedly suppressed bacterial growth, with IBPR-CuTA nearly eliminating all bacteria (Supplementary Figure S17). This superior antibacterial performance is attributed to the combined actions of the antibiotic component RF and CuTA, with RF providing sustained antibacterial protection that ensures a sterile local environment for bone regeneration.

Micro-CT evaluation of bone healing over 2–4 weeks revealed that persistent infection in the control group led to severe bone resorption and an enlarged defect, whereas the IBPR and IBPR-CuTA groups exhibited significantly better bone repair. Notably, by week 4, the IBPR-CuTA group achieved nearly complete defect regeneration, highlighting its exceptional bone-regenerative capacity (Figure 9B). Consistent with these observations, bone morphometric analysis showed that Tb.BV/TV and BMD were significantly increased in the IBPR and IBPR-CuTA groups, with the greatest improvements in the IBPR-CuTA group (Figure 9C). In addition, the IBPR-CuTA group had significantly greater trabecular thickness (Tb. Th) and lower trabecular separation (Tb. Sp) compared to the control and IBP groups (Supplementary Figure S18).

Histological analyses by hematoxylin and eosin (H&E) staining and Masson’s trichrome staining further corroborated these findings. At 2 weeks, the control defects were filled with fibrous tissue and exhibited extensive inflammatory infiltration, whereas in the IBPR and IBPR-CuTA groups, the infection had been largely eliminated and signs of new tissue formation were evident. Notably, the IBPR-CuTA defects were almost filled with newly formed collagenous matrix by this time. By 4 weeks, the control group still displayed prominent fibrous encapsulation and inflammation with no sign of new bone (consistent with micro-CT results). In the IBP and IBPR groups, the defect areas were progressively filled with collagenous tissue but showed minimal new bone formation. In stark contrast, the IBPR-CuTA group exhibited substantial new bone within the defect that closely resembled the structure of native bone (Figure 9D and E). These results demonstrated that IBPR-CuTA effectively eradicated infection and promoted robust bone regeneration in infected bone defects.

Furthermore, a critical consideration for the therapeutic application of copper-containing biomaterials is the balance between their beneficial antimicrobial effects and their potential biosafety concerns [97]. To address this, histopathological examination of the heart, liver, spleen, lungs and kidneys using H&E staining revealed no pathological abnormalities or inflammatory phenomena in any of the groups, including IBPR-CuTA, throughout the entire experimental period (Supplementary Figure S19). Notably, even after experiencing the rapid release of Cu^2+^ in an early inflammatory environment, IBPR-CuTA did not induce any copper-related toxicity. These findings collectively confirm that the IBPR-CuTA design effectively manages the inherent double-edged sword of copper, fully leveraging its therapeutic potential while proactively mitigating toxicity concerns.

**Figure 10:**
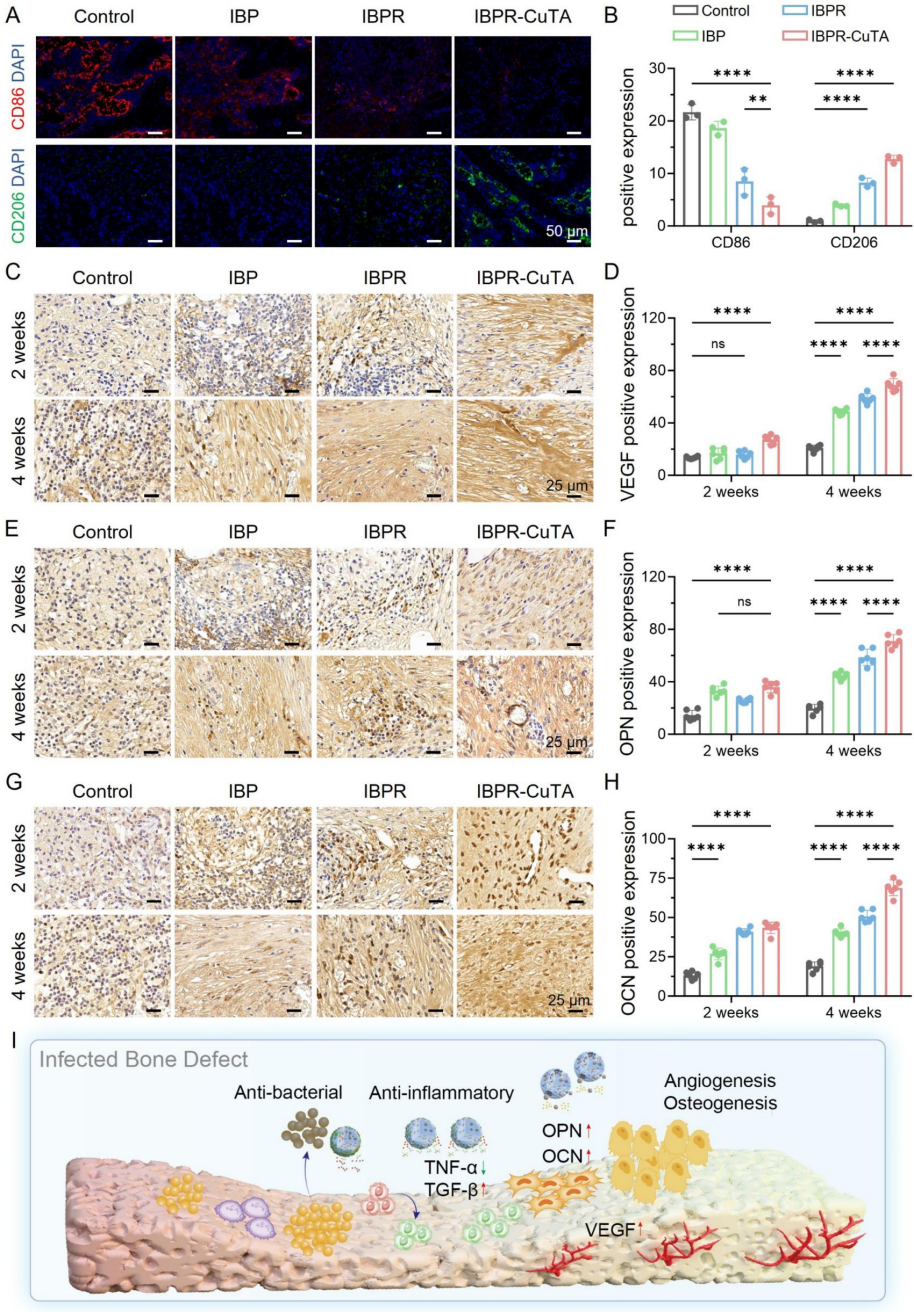
*In vivo* immunostaining analysis of IBPR-CuTA in an infected bone defect model. (**A**) Representative immunofluorescence images of CD86 and CD206 at 2 weeks post-surgery. (**B**) Quantitative analysis of CD86 and CD206-positive expression. (**C**) Representative immunohistochemical images of VEGF at 2 and 4 weeks post-surgery. (**D**) Quantitative analysis of VEGF-positive expression. (**E**) Representative immunohistochemical images of OPN at 2 and 4 weeks post-surgery. (**F**) Quantitative analysis of OPN-positive expression. (**G**) Representative immunohistochemical images of OCN at 2 and 4 weeks post-surgery. (**H**) Quantitative analysis of OCN-positive expression. (**I**) Schematic illustration of infected bone defect repair by IBPR‑CuTA microspheres through the integration of antibacterial, antioxidant, angiogenic and osteogenic effects. *P < 0.05, **P < 0.01, ***P < 0.001, ****P < 0.0001, *ns*, not significant.

In immunofluorescence staining, at 2 weeks, the control group exhibited abundant CD86^+^ cells, indicating that macrophages in the infected bone defect were predominantly polarized to M1. In contrast, the IBPR-CuTA group showed significantly fewer CD86^+^ macrophages alongside an abundance of CD206^+^ cells (Figure 10A and B). This suggests that IBPR-CuTA successfully induced macrophage polarization from M1 to M2. This demonstrated the immunomodulatory effect of IBPR-CuTA *in vivo*, as it promoted macrophage phenotype switching to alleviate inflammation and accelerate early tissue repair. To evaluate the angiogenic effect of IBPR-CuTA *in vivo*, we performed immunohistochemical staining for VEGF, a key factor in angiogenesis. At 2 and 4 weeks, the IBPR-CuTA group exhibited strong VEGF-positive signals, which were markedly enhanced compared to the other groups (Figure 10C and D). This suggested that IBPR-CuTA promoted angiogenesis at the implantation site, ensuring a sufficient blood supply for new bone formation and repair. Additionally, immunohistochemical staining was used to assess the expression of osteogenic markers OPN and OCN. The results showed that, compared to the control group, both the IBPR and IBPR-CuTA groups had significantly increased OPN and OCN positive signals at 2 and 4 weeks. Quantitative analysis confirmed that the IBPR-CuTA group exhibited the highest levels and significantly outperformed the other groups (Figure 10E–H). IBPR-CuTA not only promoted angiogenesis but also enhanced new bone maturation and mineralization, which further accelerated the bone defect repair process.

Furthermore, in the *in vivo* infected bone defect model, IBPR-CuTA rapidly eradicated the infection by releasing a large amount of RF and Cu^2+^ within the first day. The sustained release of RF over 28 days maintained a sterile environment and prevented reinfection. As the bacterial infection subsided, IBPR-CuTA from Day 3 onwards modulated macrophage polarization from M1 to M2, thereby promoting tissue repair. Meanwhile, the continuous release of Cu^2+^ stimulated new blood vessel formation, providing channels for metabolic exchange and nutrient delivery to the defect site. From Days 7 to 28, BCP and ICA released from IBPR-CuTA effectively promoted osteogenic differentiation and calcium deposition, which led to robust bone regeneration. Taken together, IBPR-CuTA exhibited efficient antibacterial activity, immunomodulatory capacity and the ability to promote angiogenesis and osteogenesis, thereby demonstrating multiple advantageous properties for repairing infected bone defects (Figure 10I). This synergistic effect significantly improved the overall healing outcome of the infected bone defects and further highlighted its potential value in bone regenerative therapy.

Although the multicomponent design of IBPR-CuTA enables coordinated antibacterial, immunomodulatory, angiogenic and osteogenic functions, it also increases the complexity of fabrication, quality control and translational development. In this study, the complexity was introduced according to the sequential pathological requirements of infected bone defects rather than by arbitrary component combination. Specifically, the outer CuTA coating serves as a pH-responsive interface for early antibacterial and immunomodulatory action, the RF-loaded PLGA matrix provides local antibiotic release and sustained infection suppression, and the ICA@BCP internal compartments provide osteogenic cues during later-stage regeneration. This spatially compartmentalized design is intended to reduce direct interference among different components and to support temporally regulated release. Further studies are needed to optimize the fabrication process, establish robust batch-to-batch quality-control standards, investigate possible long-term interactions among CuTA, RF, ICA, BCP and PLGA and evaluate large-scale manufacturing feasibility.

Several translational limitations should also be acknowledged. First, the multistep preparation process may complicate future scale-up production and regulatory quality control. Second, although preliminary batch-to-batch characterization showed acceptable reproducibility at the laboratory scale, more rigorous manufacturing validation will be required. Third, long-term copper release, copper biodistribution, RF-related antibiotic resistance risk, degradation-product clearance and chronic biosafety require further systematic investigation. Finally, the current small-animal infected bone defect model cannot fully reproduce the complexity of chronic clinical bone infections, and large-animal studies will be necessary before clinical translation.

## Conclusion

In summary, pitaya-inspired IBPR-CuTA microspheres featuring a compartmentalized configuration were developed to concurrently address the dual challenges of infection clearance and tissue regeneration associated with infected bone defects. These microspheres enable spatiotemporally controlled pH-responsive release of therapeutic agents, including antibacterial Cu^2+^ ions, the antibiotic RF and the osteogenic drug ICA, with the sustained release of RF ensuring prolonged antibacterial efficacy. Mechanistic studies revealed that IBPR‑CuTA microspheres reprogram the immune microenvironment by suppressing glucose metabolism and the TNF/NF‑κB signaling pathway, thereby inducing a macrophage polarization shift from the M1 to M2 phenotype. In addition, IBPR-CuTA significantly promotes angiogenesis and osteogenesis activity *in vitro*, indicating its capacity to stimulate vascularized bone regeneration. In an animal model of infected bone defects, IBPR-CuTA treatment simultaneously eradicated the infection and accelerated new bone formation, demonstrating its multifaceted therapeutic efficacy *in vivo*. Collectively, these findings demonstrate that IBPR-CuTA microspheres represent an innovative and multifunctional biomaterial strategy for treating infected bone defects, with significant potential for clinical translation in the treatment of complex bone diseases.

## Supplementary Material

rbag101_Supplementary_Data

## Data Availability

The data that support the findings of this study are available from the corresponding author upon reasonable request.
