# Supplementary material for: Pitaya‑inspired compartmentalized microspheres with natural tannic acid-copper coating orchestrate smart release of ions and multi-drugs for synergistic treatment of infected bone defects
Source: Regen Biomater. 2026 May 27;13:rbag101. doi: 10.1093/rb/rbag101 (PMC13278850; doi:10.1093/rb/rbag101)
Supplement: rbag101_Supplementary_Data [file rbag101_supplementary_data.zip › 03-Jun-2026_094809_Supplementary_File.docx]

**Supplementary File**

**Pitaya‑inspired compartmentalized microspheres with natural tannic acid-copper coating orchestrate smart release of ions and multi-drugs for synergistic treatment of infected bone defects**

Dongbiao Chang^a,b,c,d^, Zhenfan Bai^a,b^, Linke Li^a,d^, Jun Sheng^e^, Hongzhi Fang^d^, Zian Wang^a,b^, Zili Guo^a,b^, Hui Zhang^a,b,d^, Xinxi Yang^a,d^, Rui Chen^a,d^, Huan Tan^a^, Mengyuan Wang ^c*^, Jie Weng^a,b*^

*^a^ Institute of Biomedical Engineering, College of Medicine, Southwest Jiaotong University, Chengdu 610031, China*

*^b^ Key Laboratory of Advanced Technologies of Materials Ministry of Education, School of Materials Science and Engineering, Southwest Jiaotong University, Chengdu 610031, China*

*^c^ State Key Laboratory of Oral Diseases and National Center for Stomatology, National Clinical Research Center for Oral Diseases, West China Hospital of Stomatology, Sichuan University, Chengdu 610041, China.*

*^d^ The Center of Obesity and Metabolic Diseases, Department of General Surgery, The Third People’s Hospital of Chengdu & The Affiliated Hospital of Southwest Jiaotong University, Chengdu 610014, China.*

*^e^ Department of Orthopedic, The General Hospital of Western Theater Command of PLA, Chengdu 610083, China.*

*Corresponding authors.

E-mail addresses: [jweng@swjtu.edu.cn](mailto:jweng@swjtu.edu.cn) (Jie Weng), wangmengyuan@scu.edu.cn (Mengyuan Wang)

Table S1. Gene primer sequences used in this study.

| species | Gene | Forward Primer | Reverse Primer |
| --- | --- | --- | --- |
| *Mouse* | *Gapdh* | TGCACCACCAACTGCTTAG | GATGCAGGGATGATGTTC |
|  | *Tnf-α* | GAACTGGCAGAAGAGGCACT | AGGGTCTGGGCCATAGAACT |
|  | *Il-1β* | AATGCCACCTTTTGACAGTGATG | CATCTCGGAGCCTGTAGTGC |
|  | *Tgf-β* | AGGGCTACCATGCCAACTTC | CCACGTAGTAGACGATGGGC |
|  | *Il-10* | GCATGGCCCAGAAATCAAGG | CATTCATGGCCTTGTAGACACC |
| *Human* | *GAPDH* | ATGATTCCACCCATGGCAAATTC | TGGTTCACACCCATGACGAA |
|  | *VEGF* | CCACACCATCACCATCGACA | CCCTCCCAACTCAAGTCCAC |
|  | *eNOS* | CGAGTGAAGGCGACAATCCT | CGAGGGACACCACGTCATAC |
|  | *ANG-1* | TGCCATTACCAGTCAGAGGC | AGCACCGTGTAAGATCAGGC |
| *Rat* | *Gapdh* | TCAACAGCAACTCCCACTCTTCCA | ACCCTGTTGCTGTAGCCGTATTCA |
|  | *Alp* | CCAACTCTTTTGTGCCAGAGA | GGCTACATTGGTGTTGAGCTTTT |
|  | *Bmp-2* | AAGCGTCAAGCCAAACACAAACAG | CCAGTCATTCCACCCCACATCAC |
|  | *Runx2* | AACGATCTGAGATTTGTGGGC | CCTGCGTGGGATTTCTTGGTT |
|  | *Col1a1* | TGAACGTGGTGTACAAGGTC | CCATCTTTGCCAGGAGAACCAT |
|  | *Opn* | CCAGCCAAGGACCAACTACA | AGTGTTTGCTGTAATGCGCC |
|  | *Ocn* | GGCGTCCTGGAAGCCAATGTG | GACCAGGAGGACCAGGAAGTCCACGT |


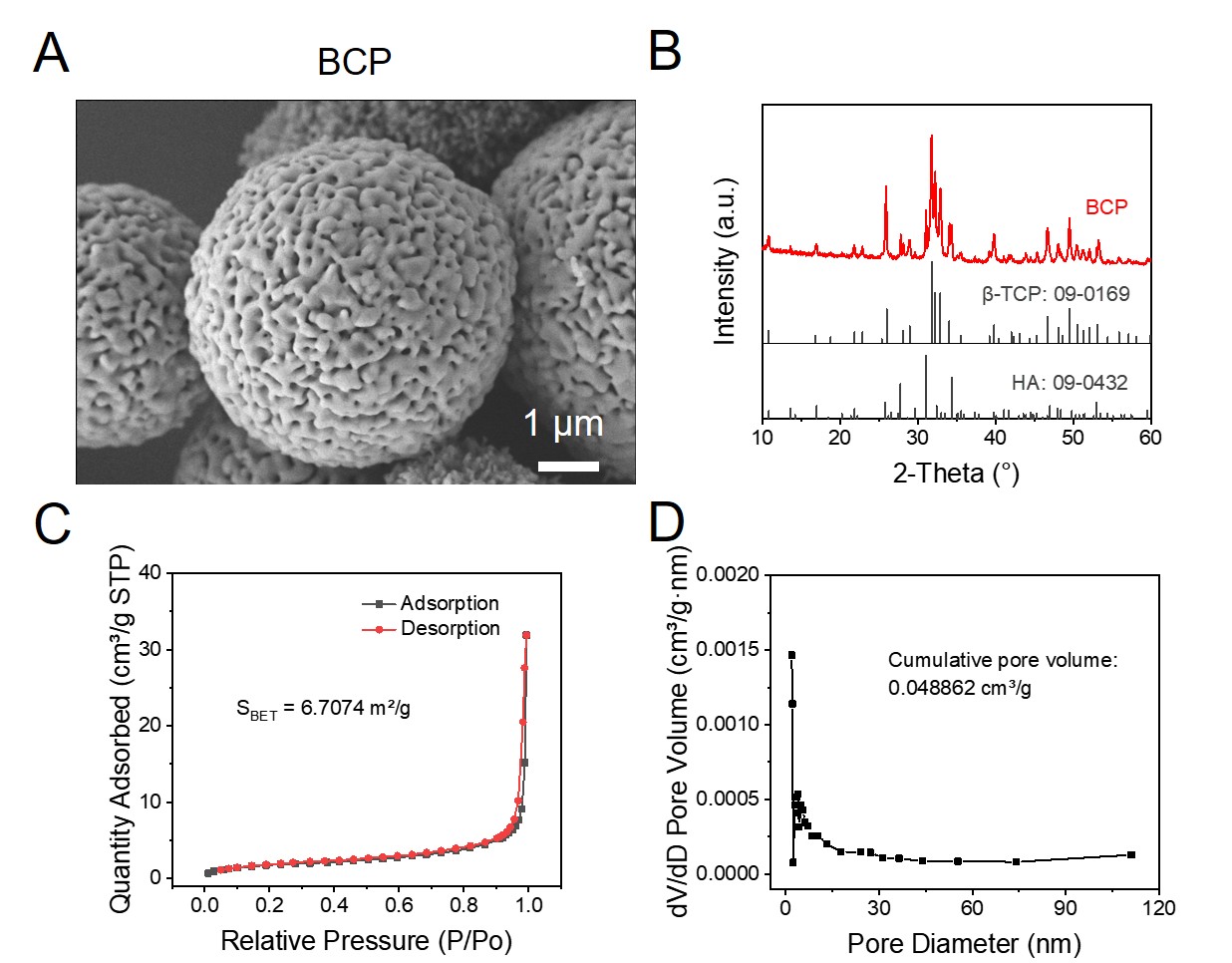


**Figure S1.** Characterization of BCP microspheres. (A) SEM images; (B) XRD pattern; (C, D) Nitrogen adsorption-desorption isotherms and pore size distribution curves.


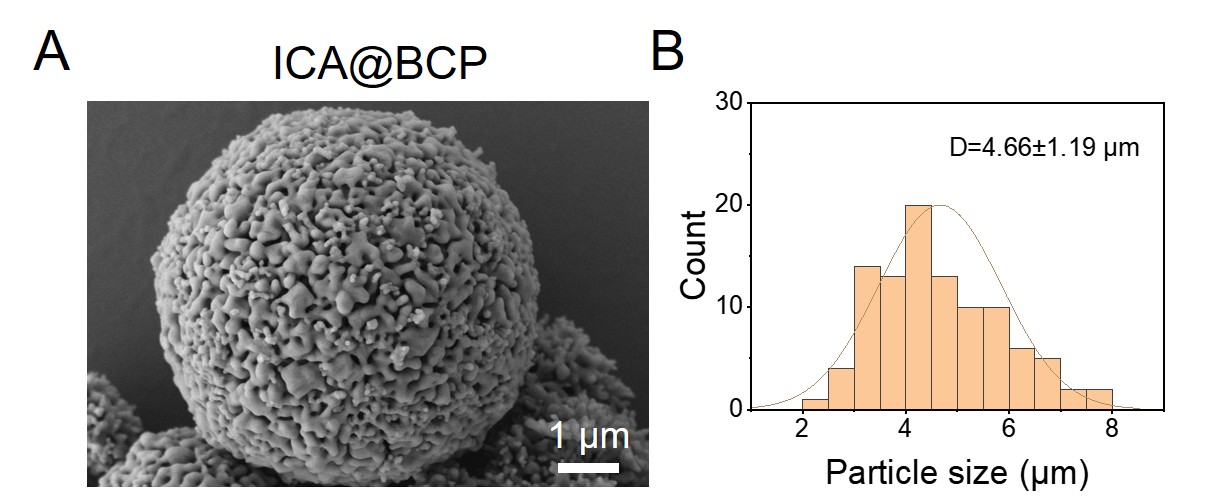


**Figure S2.** SEM image (A) and particle size distribution (B) of ICA@BCP microspheres.


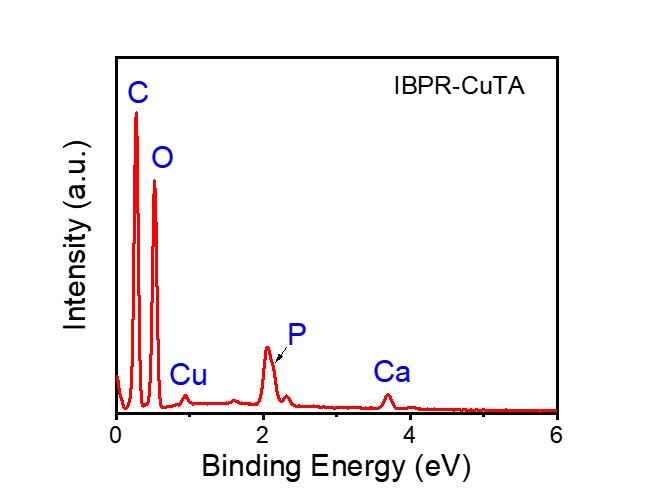


**Figure S3.** EDS spectrum of IBPR-CuTA.


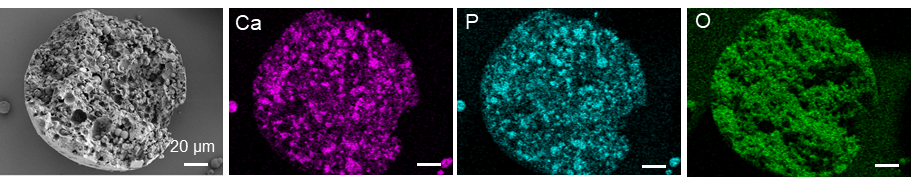


**Figure S4.** Cross-sectional SEM images and EDS elemental mapping of IBPR-CuTA.


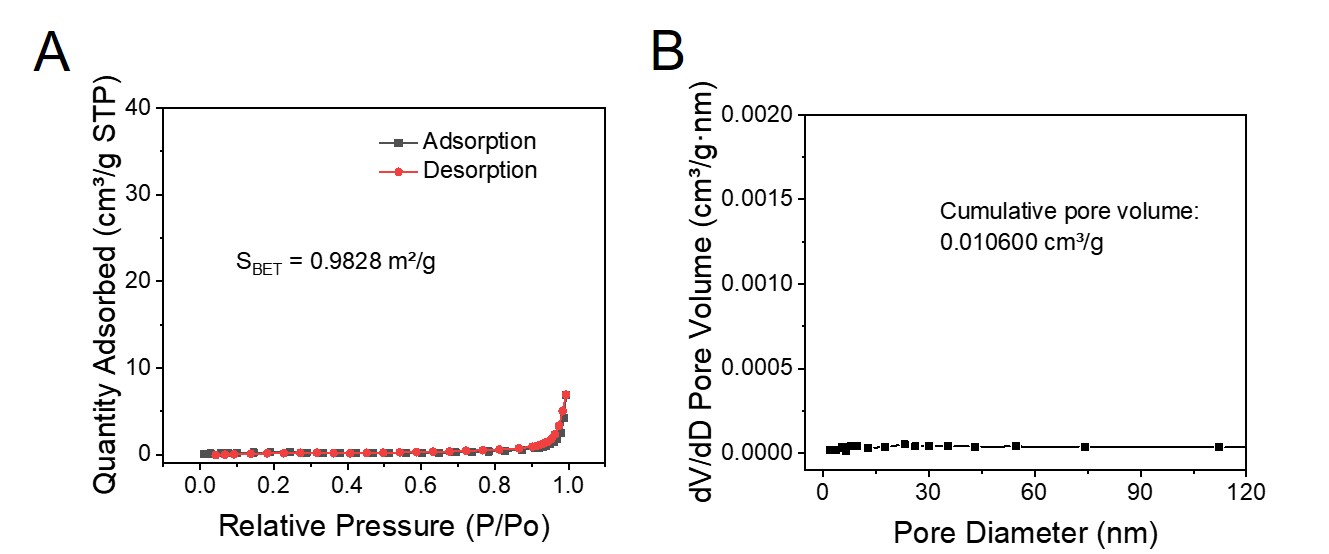


**Figure S5.** Nitrogen adsorption-desorption isotherms (A) and pore size distribution curves (B) of IBPR-CuTA microspheres.

**Figure S6.** Zeta potential of IBPR-CuTA microspheres.


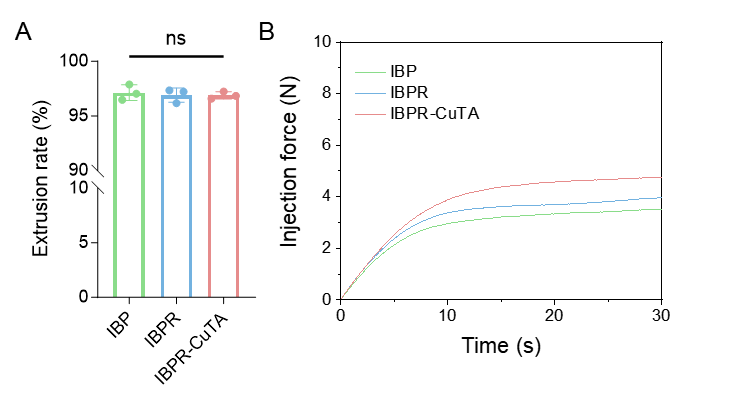


**Figure S7.** The extrusion rate (A) and injection force (B) of IBPR‑CuTA microspheres.


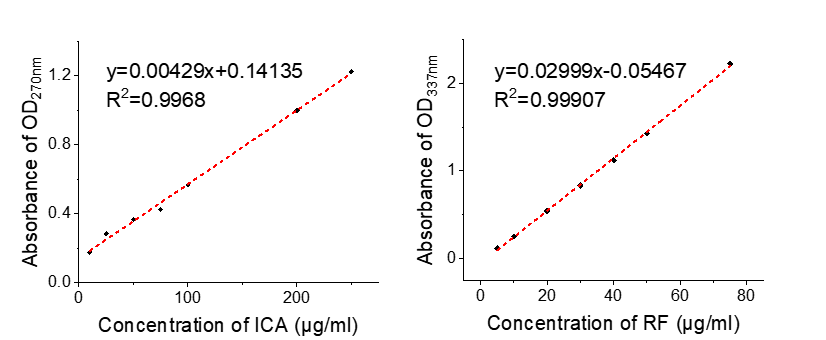


**Figure S8.** Standard curves of ICA and RF absorbance at varying concentrations.


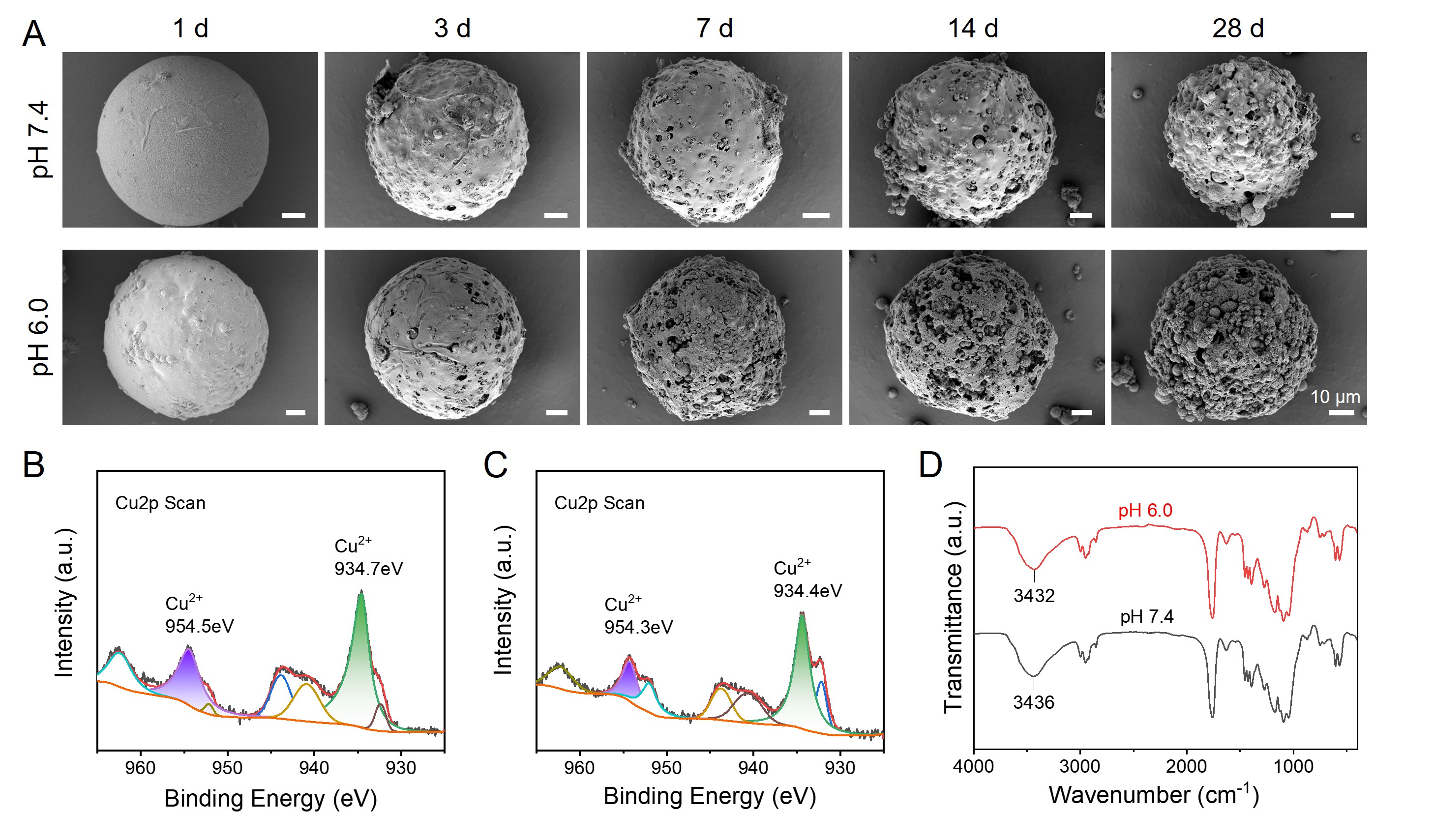


**Figure S9.** (A) SEM images of IBPR-CuTA microspheres after 28 days of degradation in PBS at different pH values; (B, C) High-resolution Cu 2p XPS spectra of IBPR-CuTA microspheres after 1 day incubation in PBS at pH 7.4 (B) and pH 6.0 (C); (D) FTIR spectra of IBPR-CuTA microspheres after 1 day incubation in PBS at different pH values.


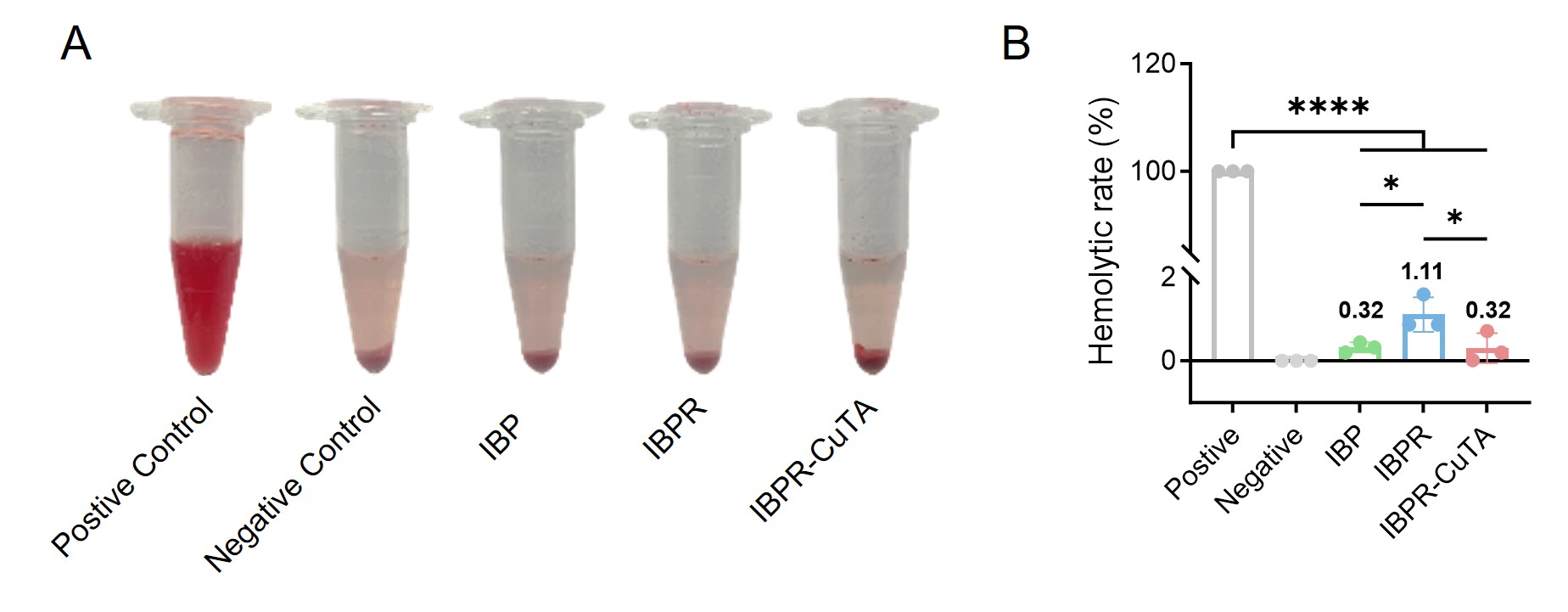


**Figure S10.** Hemocompatibility of IBPR-CuTA. (A) Photographs of hemolysis assay; (B) hemolysis ratio. Data are presented as mean ± standard deviation (n = 3), **P* < 0.05, ***P* < 0.01, ****P* < 0.001, *****P* < 0.0001, *ns*: no significant difference.


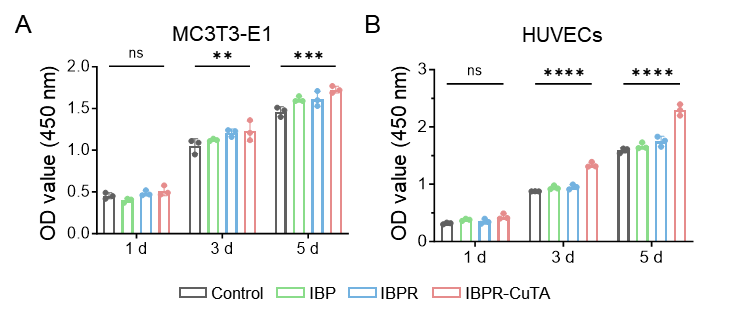


**Figure S11.** Cell proliferation activity. OD values of MC3T3-E1 cells (A) and HUVECs (B) cultured with materials for 1, 3, and 5 days. Data are presented as mean ± standard deviation (n = 3), **P* < 0.05, ***P* < 0.01, ****P* < 0.001, *****P* < 0.0001, *ns*: no significant difference.


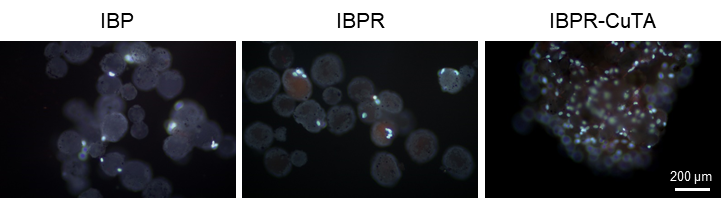


**Figure S12.** Fluorescence images of DAPI-stained cell nuclei of MC3T3-E1 after co-culturing with microspheres for 3 days.


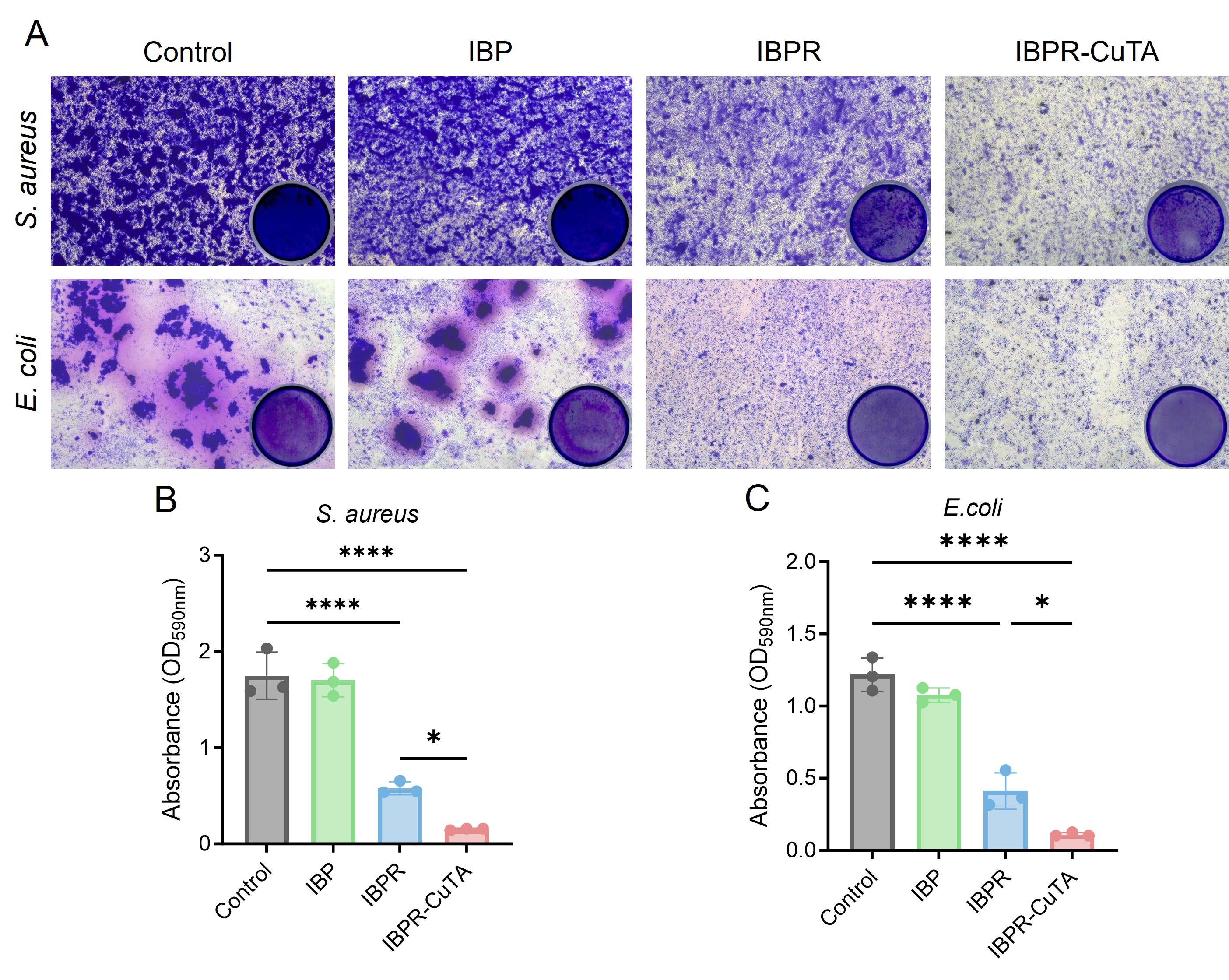


**Figure S13.** Antibiofilm activity of IBPR-CuTA. (A) Crystal violet staining images; (B, C) Absorbance at 590 nm for S. aureus and E. coli after elution of crystal violet stain.


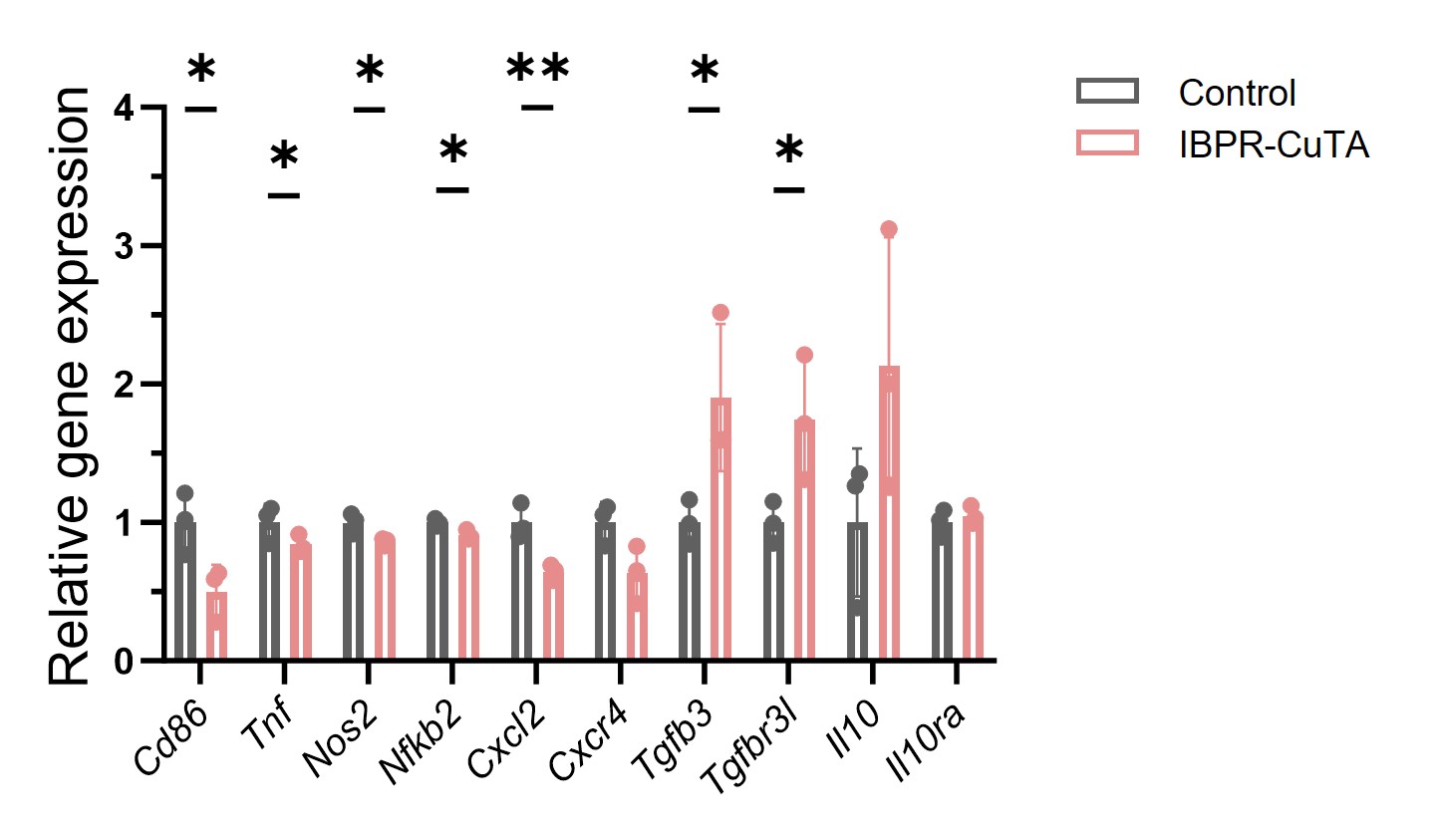


**Figure S14.** Relative expression levels of representative genes from the transcriptome sequencing gene list. Data are presented as mean ± standard deviation (n = 3), **P* < 0.05, ***P* < 0.01, *ns*: no significant difference.


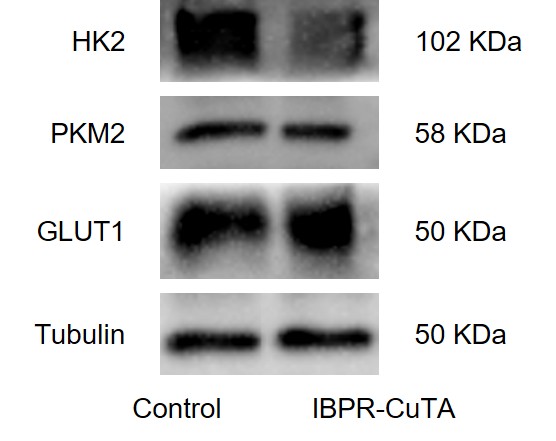


**Figure S15.** Western blot analysis of key glucose metabolism protein expression (GLUT1, PKM2, HK2) in RAW264.7 cells


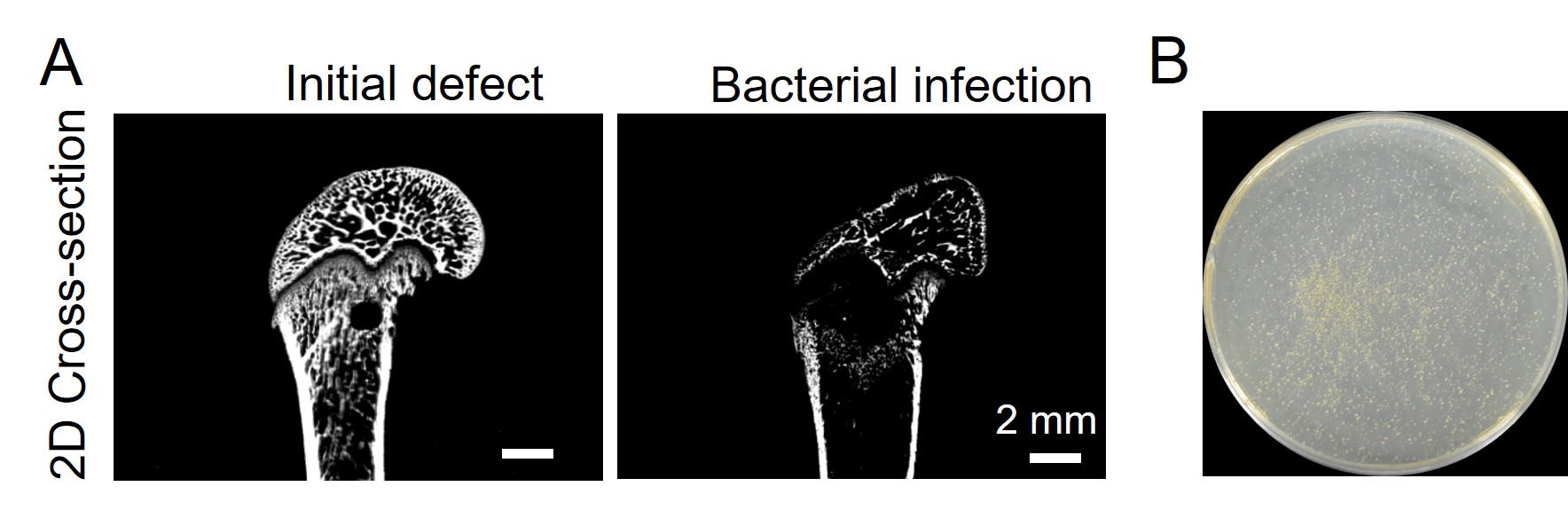


**Figure S16.** Evaluation of the infectious bone defect model. (A) 2-dimensional micro-CT sections of the femur 1 week post-modeling. (B) digital photographs of colony plating after 24 h incubation.


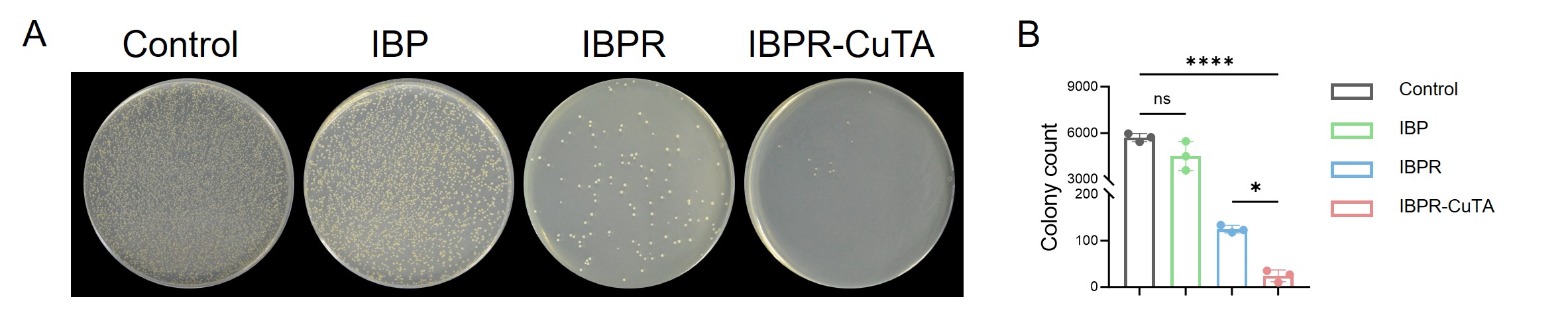


**Figure S17.** In vivo antibacterial performance of IBPR-CuTA. (A) Bacterial plating of bone tissue and (B) colony quantification at 2 weeks post-surgery in the infectious bone defect model. Data are presented as mean ± standard deviation (n = 3), **P* < 0.05, ***P* < 0.01, ****P* < 0.001, *****P* < 0.0001, *ns*: no significant difference.


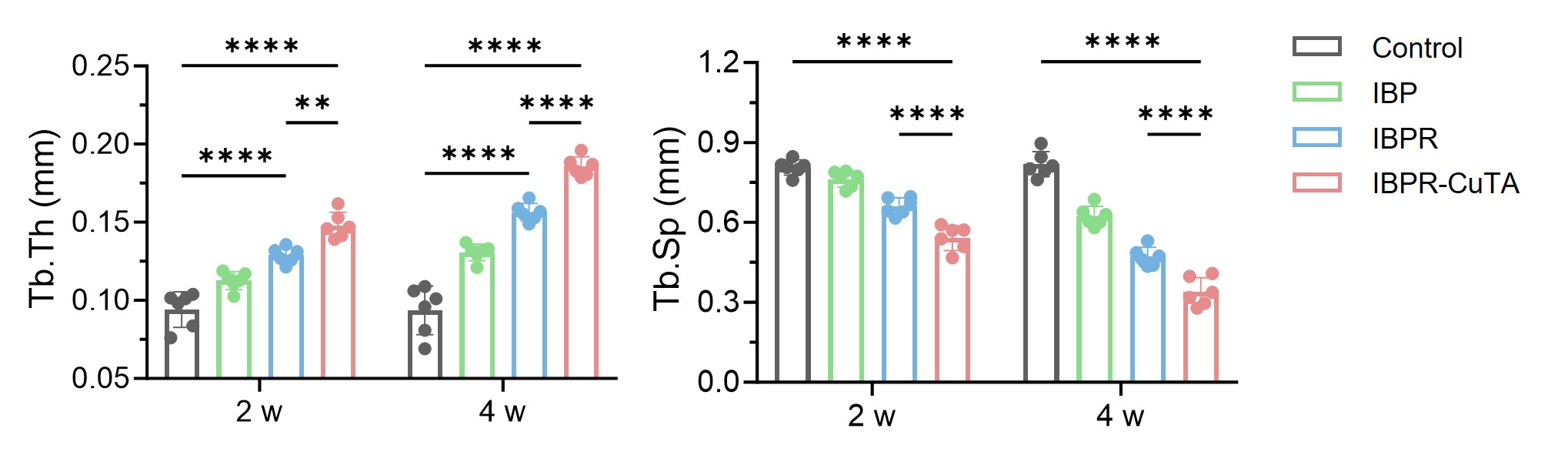


**Figure S18.** Trabecular thickness (Tb.Th) and trabecular separation (Tb.Sp) of bone defects at 2 and 4 weeks after implantation. Data are presented as mean ± standard deviation (n = 6), **P* < 0.05, ***P* < 0.01, ****P* < 0.001, *****P* < 0.0001, *ns*: no significant difference.


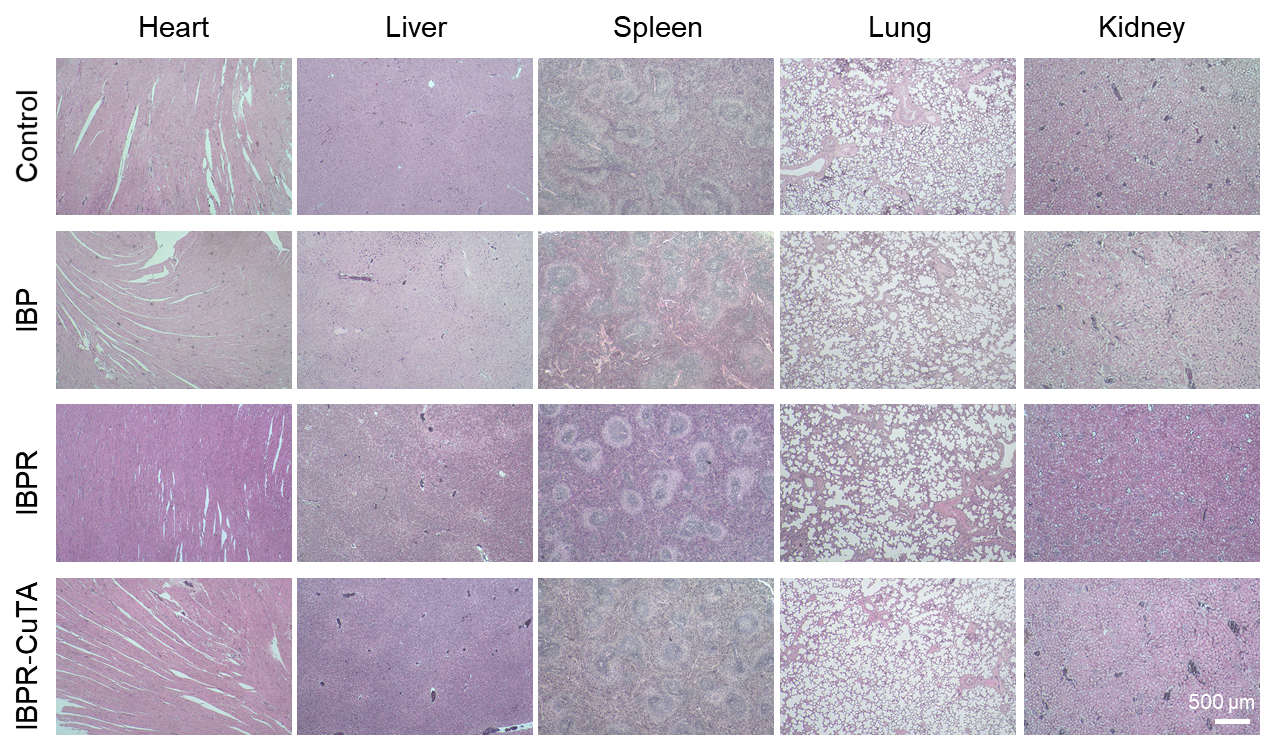


**Figure S19.** H&E staining of major organs (heart, liver, spleen, lung, kidney) at 4 weeks after implantation in each group.
